# The modulatory effects of alfalfa polysaccharide on intestinal microbiota and systemic health of *Salmonella serotype (ser.) Enteritidis*-challenged broilers

**DOI:** 10.1038/s41598-021-90060-6

**Published:** 2021-05-25

**Authors:** Zemin Li, Chongyu Zhang, Bo Li, Shimin Zhang, Fawaz G. Haj, Guiguo Zhang, Yunkyoung Lee

**Affiliations:** 1 Department of Animal Nutrition, Shandong Agricultural University, 61 Daizong Street, Taian City, 271018 China; 2 Department of Nutrition, University of California Davis, One Shields Ave, Davis, CA 95616 USA; 3 Department of Food Science and Nutrition, and Interdisciplinary Graduate Program in Advanced Convergence Technology and Science, Jeju National University, Jeju, 63243 South Korea

**Keywords:** Biological techniques, Microbiology

## Abstract

*Salmonella serotype (ser.) Enteritidis* infection in broilers is a main foodborne illness that substantially threatens food security. This study aimed to examine the effects of a novel polysaccharide isolated from alfalfa (APS) on the intestinal microbiome and systemic health of *S. ser. Enteritidis-*infected broilers. The results indicated that broilers receiving the APS-supplemented diet had the improved (*P* < 0.05) growth performance and gut health than those fed no APS-supplemented diet. Supplementation with APS enhanced (*P* < 0.05) the richness of gut beneficial microbes such as *Bacteroidetes*, *Barnesiella*, *Parabacteroides*, *Butyricimonas*, and *Prevotellaceae*, while decreased (*P* < 0.05) the abundance of facultative anaerobic bacteria including *Proteobacteri*a, *Actinobacteria*, *Ruminococcaceae*, *Lachnospiraceae*, and *Burkholderiaceae* in the *S. ser. Enteritidis*-infected broilers. The *Bacteroides* and *Odoribacter* were identified as the two core microbes across all treatments and combined with their syntrophic microbes formed the hub in co-occurrence networks linking microbiome structure to performance of broilers. Taken together, dietary APS supplementation improved the systemic health of broilers by reshaping the intestinal microbiome regardless of whether *S. ser. Enteritidis* infection was present. Therefore, APS can be employed as a potential functional additives to inhibit the *S. ser. Enteritidis* and enhance the food safety in poultry farming.

## Introduction

*Salmonella serotype (ser.) Enteritidis* is a well-known foodborne pathogen throughout the world^1,2^. Consumption of *S. ser. Enteritidis*-contaminated chicken and eggs was the major cause of human salmonellosis, and contaminated products can all be traced to infected poultry in breeding facilities^3,4^. Although *S. ser. Enteritidis*-challenged broilers exhibited low mortality rates, the persistent colonization of *S. ser. Enteritidis* in broilers resulted in environmental pollution and posed the most severe threats to food security and public health^1,5^. Additionally, *S. ser. Enteritidis* can effectively adjust to environmental changes to successfully survive in the gut or environment^1,6^. Given its ubiquity, it was unlikely that *S. ser. Enteritidis* will be eradicated from the food chain, but it is feasible to find intervention strategies to inhibit its colonization in animal intestines and to control its spread in the food chain, thus improving animal health and food safety^1,7,8^. Previous studies have documented that *S. ser. Enteritidis* in the gastrointestinal tract interfered with optimal nutrient metabolism and immune function and thus subsequently retarded the growth of broilers^9,10^. Antibiotics have been applied as means to eliminate damage in the intestinal tract caused by *S. ser. Enteritidis* infection; however, their use has raised even more concerns, regarding the possible presence of drug residues in meats^6,11,12^. Therefore, how to eliminate or mitigate the damage caused by the *S. ser. Enteritidis* infection and inhibit its colonization in the gut has attracted increasing attention^1,10,13^. Currently, a reasonable body of evidences support the notion that certain polysaccharides could be added to broiler’s diet to eliminate and/or mitigate the damage caused by *S. ser. Enteritidis* infection. Parsons and Wigley (2014) found that a polysaccharide from plantain prevented *S. ser. Enteritidis* from adhering to or translocating across the animal intestinal epithelium and thus reduced intestinal damage. Similarly, a novel polysaccharide isolated from *Dictyophora indusiate* was documented to promote the recovery from antibiotic-driven intestinal dysbiosis and improve gut epithelial barrier function in a mouse model^14^. In previous study, we have deciphered that APS was composed of 7 monosaccharides and 2 uronic acids with the molar mass of 3.3 × 10^6^ D, and specially stimulated B cells proliferation^15^. In another parallel trial, APS exhibited superior antioxidant and anti-inflammatory bioactivity by activating MAPK/Nrf2 and suppressing NF-κB signaling pathways^16^. Additionally, beneficial effects of alfalfa polysaccharide (APS) supplementation in piglet diets have also demonstrated, as inclusion of APS in diet increased intestinal beneficial bacteria proliferation and improved intestinal morphology by increasing villus height and decreasing crypt depth, thus helping to enhance average daily growth (ADG) and feed conversion rates (FCR)^17^. However, the effects of dietary supplementation with APS on the performance, intestinal, and intestinal microbiota of *S. ser. Enteritidis*-infected broilers remains unclear.

It was hypothesized that dietary APS supplementation would alter the microbiota composition in terms of microbial diversity and abundance and improve systemic health in *S. ser. Enteritidis*-infected broilers, thus exerting a beneficial effect on production performance. The objectives of this study were to investigate (1) the effects of dietary supplementation with APS in *S. ser. Enteritidis*-challenged broiler diet on intestinal microbial diversity and abundance, (2) the effects of dietary APS supplementation on the intestinal health, immune status, and performance of *S. ser. Enteritidis*-challenged broilers.

## Results

### Beneficial effects of APS on the growth and immune status of broilers

**Figure 1:**
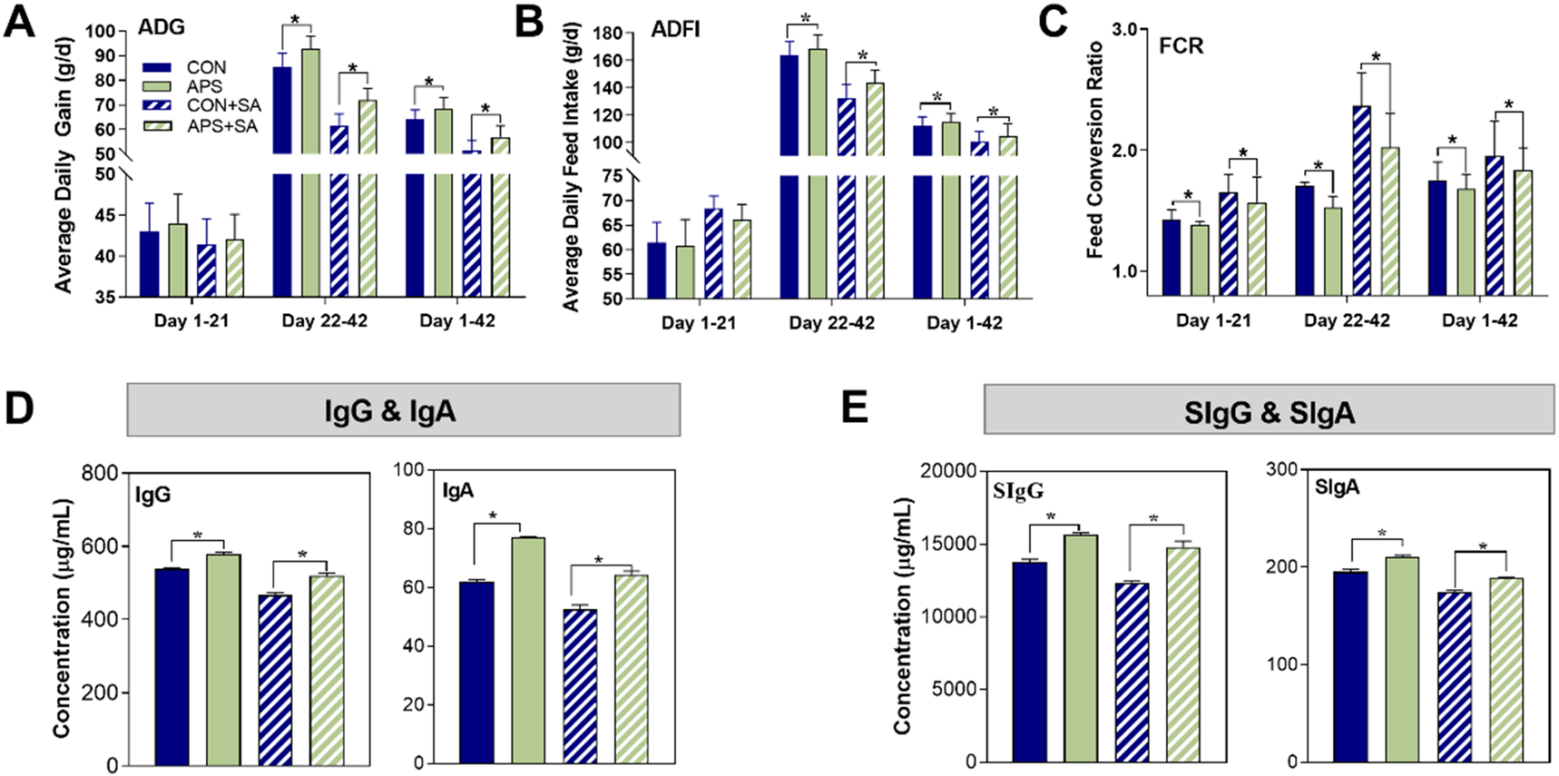
Effects of APS supplementation on growth performance and immune status in pair-fed and *S. ser. Enteritidis*-challenged broilers. (**A**) Average daily gain (ADG), (**B**) average daily feed intake (ADFI), (**C**) feed conversion ratio (FCR, calculated by ADFI/ADG),) (**D**) IgG and IgA content in serum, and **E**. SIgG and SIgA content in the duodenal mucosa of broilers. The data are expressed as the mean ± SEM, and an asterisk (*) indicated a significant difference by one-way ANOVA (*p* < 0.05).

The effects of APS supplementation on ADG, average daily feed intake (ADFI), and FCR, were determined on broilers in both pair-fed groups (the control (CON) and APS groups) and *S. ser. Enteritidis*-challenged groups (the CON + *S. ser. Enteritidis VS.* APS + *S. ser. Enteritidis* groups, abbreviated as CON + SA *VS.* APS + SA) (Supplementary Fig. S1A). An interesting finding of this study was that, regardless of the whole experimental period or certain growing stages (1 to 21 days, or 22 to 42 days), the APS-supplemented group had an increased ADG, yet decreased ADFI and FCR compared with that of the CON group. Similarly, in the case of *S. ser. Enteritidis*-infection, APS supplementation (APS + SA) augmented the ADG while reduced the ADFI and FCR of broilers than that un-supplemented group (CON + SA) (Fig. 1A–C). As shown in Fig. 1D, the APS-supplemented diet increased (*P* < 0.05) serum IgG and IgA levels compared to the control diet in both the pair-fed and *S. ser. Enteritidis*-challenged groups. Similar alterations were also observed for increased SIgA and SIgG contents (Fig. 1E) in the duodenal mucosa, suggesting that APS supplementation improved immune status of broilers.

### Effects of APS supplementation on intestinal health

**Figure 2:**
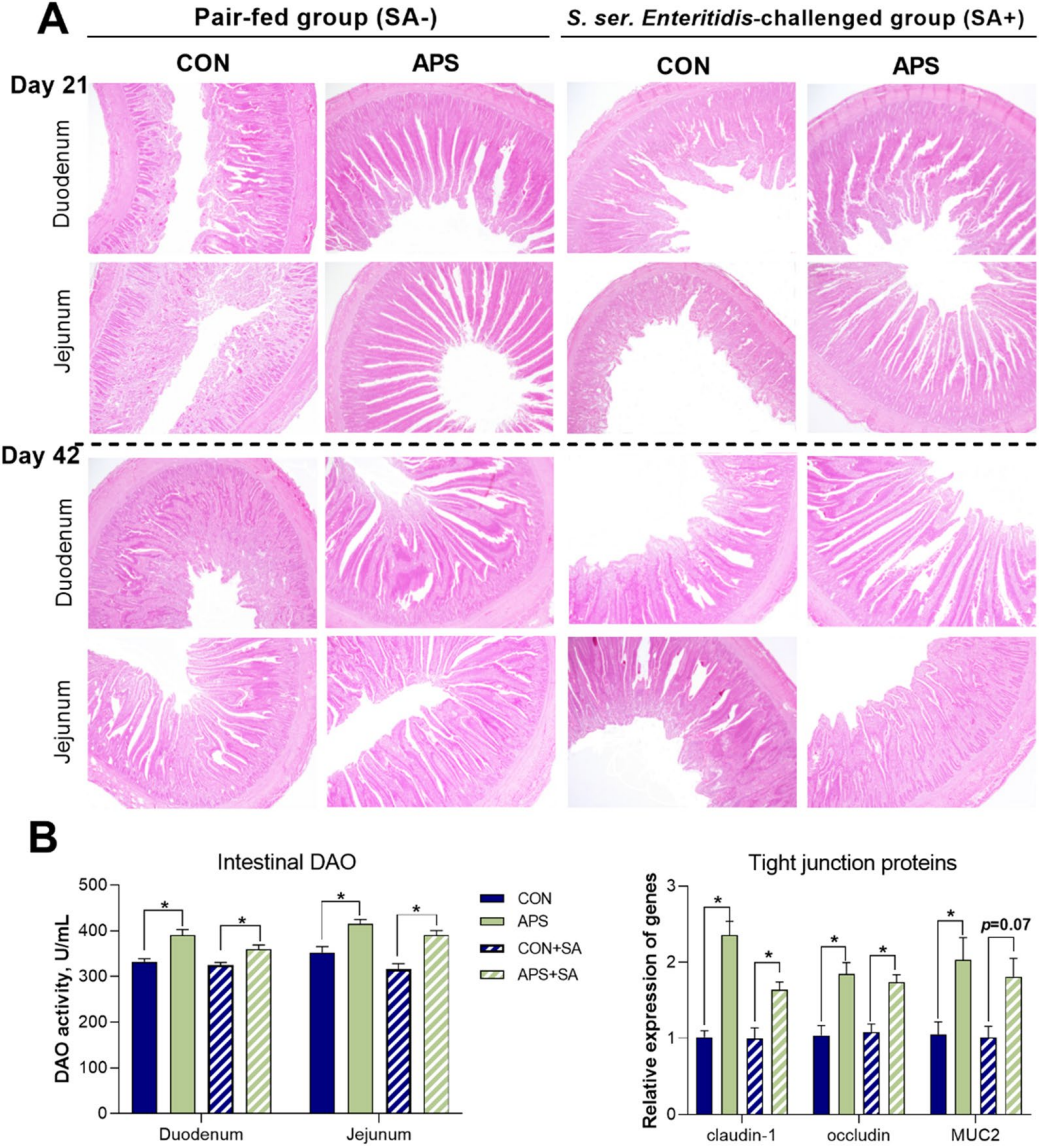
Effect of APS supplementation on gut development, intestinal mucosa enzyme activity and tight junction-related protein mRNA expression in pair-fed vs. *S. ser. Enteritidis*-challenged groups. (**A**) Hematoxylin and eosin (HE) staining of the duodenum and jejunum on days 21 and 42 in the four experimental groups (n = 6). The villus and crypts from each segment were measured with a light microscope equipped with Image-Pro Plus software (version 6.0, Motic Images software, Motic China Group Co., Ltd., Xiamen, China, https://www.semi.org/en/resources/member-directory/motic-china-group-co-ltd) and stained with HE, × 100 magnification, (**B**) The diamine oxidase (DAO) activity in the duodenal and jejunal mucosa and mRNA expression of tight junction-related proteins in the jejunum, including claudin-1, occludin, and MUC-2 (n = 6).

Next, we investigated how APS supplementation affected intestinal development and barrier functions in broilers at two time points (day 21 and day 42) by analyzing histological alterations in the duodenum and jejunum, the activity of diamine oxidase (DAO) and the relative mRNA expression of tight junction (TJ)-related proteins (Fig. 2 & Table 1). The rsults revealed that APS group exhibited a significantly greater (*P* < 0.05) villus height than the CON group and that the APS + SA group exhibited a significantly shallower crypt depth than the CON + SA group. These changes significantly increased (*P* < 0.05) the villus/crypt (V/C) ratios in both groups of APS-treated broilers compared with their corresponding control groups (Fig. 2A & Table 1). With regard to the development of the jejunum on day 21, we similarly observed significantly increased (*P* < 0.05) V/C ratios in the APS-supplemented groups in both the pair-fed and *S. ser. Enteritidis*-challenged conditions. Similarly, the APS-supplemented groups consistently displayed improvements in gut villus development in the duodenal and jejunal on day 42.

**Table 1:** Effects of supplementing alfalfa polysaccharide (APS) to *S. ser. Enteritidis*-challenged broilers on the small intestinal morphology.

Items		Pair-fed groups (SA-)		*S. ser. Enteritidis*-challenged groups (SA +)	
		CON	APS	CON	APS
**Day 21**					
Duodenum	VH^1^	1170.200 ± 57.669^b^	1483.600 ± 101.750^a^	1410.180 ± 36.759^a^	1393.100 ± 49.587^a^
	CD	278.620 ± 14.744^c^	303.000 ± 19.789^bc^	363.400 ± 19.253a	313.400 ± 28.987^b^
	V/C	4.204 ± 0.174^b^	4.900 ± 0.240^a^	3.888 ± 0.192^c^	4.465 ± 0.281^b^
Jejunum	VH	1298.040 ± 82.939^b^	1372.880 ± 20.366^a^	636.920 ± 63.336^c^	1379.460 ± 67.106^a^
	CD	316.920 ± 29.990a	282.920 ± 32.330b	168.500 ± 11.765c	311.720 ± 10.946ab
	V/C	4.109 ± 0.238bc	4.896 ± 0.482a	3.781 ± 0.289c	4.432 ± 0.301b
**Day 42**					
Duodenum	VH	1241.684 ± 207.218c	1388.398 ± 168.999b	1121.108 ± 69.951c	1572.710 ± 141.133a
	CD	282.006 ± 60.378b	248.926 ± 25.752c	301.846 ± 22.934ab	325.162 ± 35.160^a^
	V/C	4.442 ± 0.295^b^	5.593 ± 0.553^a^	3.721 ± 0.181^c^	4.853 ± 0.318^b^
Jejunum	VH	1195.558 ± 40.631^b^	1433.746 ± 107.729^a^	1152.476 ± 53.036^b^	1264.282 ± 99.474^b^
	CD	270.430 ± 21.003^ab^	235.906 ± 14.752^c^	285.592 ± 18.375^a^	249.478 ± 28.949^bc^
	V/C	4.438 ± 0.307^c^	6.075 ± 0.161^a^	4.046 ± 0.269^c^	5.098 ± 0.468^b^

^a-c^Different letters in the same row indicate significant differences (*P* < 0.05), and the same letters mean no significant difference (*P* > 0.05).

^1^VH, villus height. CD, crypt depth. V/C, the ratio of Villus height to the crypt depth.

Consistent with the intestinal histology alterations, the activities of the gut mucosa DAO and the relative mRNA expression of the TJ-related proteins involving claudin-1, occludin, and MUC-2 were significantly increased (*P* < 0.05) in the APS-supplemented groups compared to the control groups regardless of whether the broilers were subjected to *S. ser. Enteritidis* challenge (Fig. 2B). This revealed ameliorated intestinal barrier function due to the supplementation of APS.

## Summary of cecal microbial community richness and diversity in broilers

The microbiota of the cecal contents of the broilers in the four experimental groups (CON, APS, CON + SA, and APS + SA) was analyzed by sequencing of the bacterial 16S rDNA V3 + V4 region. High-throughput pyrosequencing of the cecum samples (n = 6/group) generated a total of 2,791,470 raw reads. After low-quality sequences were removed, 2,132,125 clean reads (Total Tag) for the cecum were obtained. Based on a threshold of 97% sequence similarity, a total of 3,064, 2,799, 2,868, and 3,000 operational taxonomic units (OTUs) were identified in the cecal content samples of the CON, APS, CON + SA, and APS + SA groups, respectively (Supplementary Table S2).

**Figure 3:**
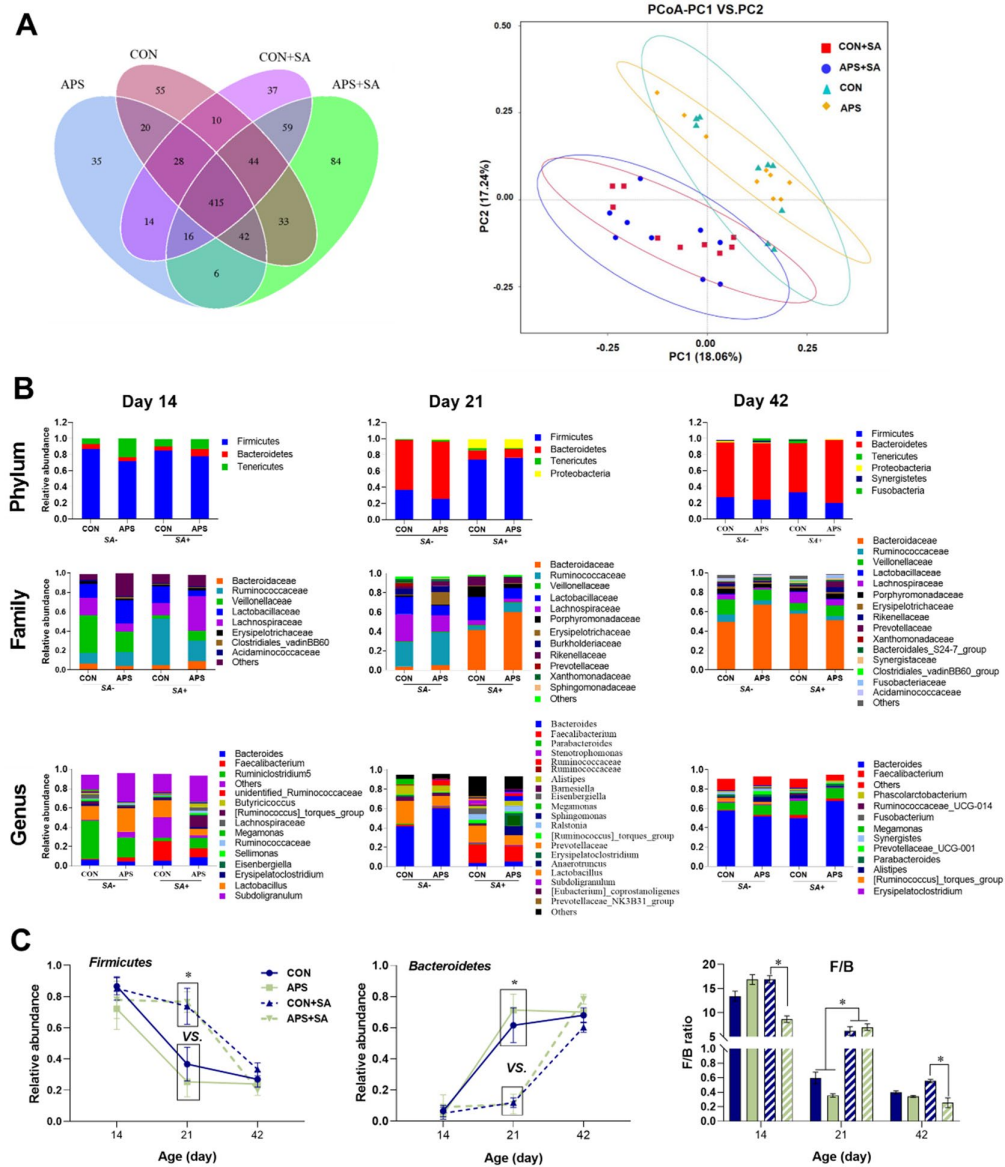
The OTU numbers and relative abundance values in the cecal microbiota at the phylum, family, and genus levels. (**A**) OTU number Venn plot and PCoA score plot for the different groups, (**B**) Relative abundance values in the cecal microbiota of broilers at 14, 21 and 42 days of age at the phylum, family, and genus levels, (**C**) Changes in the abundance values of the dominant phyla *Firmicutes* and *Bacteroidetes* and their ratio (F/B) in different ages of broilers. Only microbes that had a mean relative abundance greater than 1% are displayed in this figure. CON, control pair-fed group; APS, APS-supplemented pair-fed group; CON + SA, control *S. ser. Enteritidis*-challenged group; APS + SA, APS-supplemented *S. ser. Enteritidis*-challenged group; *SA* + , *S. ser. Enteritidis*-challenged groups; *SA-*, pair-fed groups.

The sequencing depth reflected the total microbial species richness (good coverage > 99%), and the majority of OTUs presented low abundance, and there were no significant difference (*P* > 0.05) in alpha-diversity among all groups, as demonstrated by the rarefaction curve, rank abundance, Shannon index, and phylogenetic tree (PD_whole_tree) (Table 2, Supplementary Fig. S2). The average OTU numbers were 325, 405, and 647 in the CON group at 14, 21, and 42 days of age, respectively. Similarly, there were 309, 456, and 434 OTUs in the APS group; 361, 482, and 431 OTUs in the CON + SA group; and 419, 408, and 699 OTUs in the APS + SA group at 14, 21, and 42 days of age, respectively (Supplementary Fig. S3). However, the shared OTU numbers across the four experimental groups increased (*P* < 0.05) with the broilers’ age with increasing time post *S. ser. Enteritidis* infection. In general, the OTU numbers of the APS + SA group were significantly greater (*P* < 0.05) than those of the APS group at 14 and 42 days of age, while there were similar OTU numbers among all groups at 21 days of age (*P* = 0.188) and among the pooled samples regardless of age (*P* = 0.591). To analyze the β-diversities of the cecal samples, the unweighted Unifrac distances were compared among the four different groups. The microbial community structures in the CON, APS, CON + SA, and APS + SA groups at different stages of development (14, 21, and 42 days of age) were almost separated in the hierarchical clustering tree in an age-dependent manner; in addition, principal coordinate analysis (PCoA) indicated that the microbial communities were clearly different between the *S. ser. Enteritidis*-infected and pair-fed groups (Fig. 3A, Supplementary Fig. S4).

**Table 2:** Alpha diversity indices of the cecal microbiota of broilers.

Group^1^	Coverage, %	Richness estimator			Diversity index	
		Chao1	ACE	PD_whole_tree	Shannon	Simpson
CON	> 99	362.45 ± 20.02	369.9 ± 21.96	21.84 ± 1.61	4.41 ± 0.21	0.864 ± 0.023
APS	> 99	347.25 ± 26.87	349.67 ± 25.83	19.8 ± 1.26	4.02 ± 0.28	0.827 ± 0.034
CON + SA	> 99	361.53 ± 29.60	364.36 ± 28.79	23.02 ± 1.21	4.34 ± 0.18	0.868 ± 0.017
APS + SA	> 99	406.69 ± 38.84	388.8 ± 28.67	24.23 ± 1.43	4.41 ± 0.17	0.881 ± 0.014

^1^CON: control pair-fed group; APS: APS-supplemented pair-fed group; CON + SA: control *S. ser. Enteritidis*-challenged group; APS + SA: APS-supplemented *S. ser. Enteritidis*-challenged group.

### Characteristics of the cecal microbiota in broilers received different treatments

The relative abundance of cecal microbes (> 1%) in broilers of different ages was determined at the phylum, family, and genus levels (Fig. 3B). The cecal microbiota was dominated by the phyla *Firmicutes* and *Bacteroidetes* in all groups. Regardless of treatment, the richness of Bacteroidetes persistently increased, while the abundance of *Firmicutes* and the ratio of *Firmicutes* to *Bacteroidetes* (the F/B ratio) decreased, with increasing broiler age. However, the richness of *Bacteroidetes* and *Firmicutes* altered to a less extent for CON + SA and APS + SA groups than that of CON and APS groups on day 21.

At 14 days of age (the first administration of *S. ser. Enteritidis*), the proportions of the phylum *Firmicutes* (regardless of treatment) were 86.66%, 72.10%, 85.21%, and 77.95% in the CON, APS, CON + SA, and APS + SA groups, respectively, while the *Bacteroidetes* richness accounted for 6.45%, 4.27%, 5.04%, and 8.99% of the total abundance, respectively (Fig. 3B, Supplementary Table S2). APS supplementation decreased (*P* < 0.05) the ratio of *Firmicutes* to *Bacteroidetes* (F/B values). At 21 days of age, the abundance of *Firmicutes* was similar in the CON + SA (73.80%) and APS + SA (76.57%) groups and was higher than that in the CON (36.68%) and APS (25.31%) groups. In contrast, the richness of the phylum *Bacteroidetes* in the CON (61.57%) and APS (71.26%) groups was higher than that in the CON + SA (11.87%) and APS + SA (10.92%) groups (*P* = 0.034). The F/B values of the CON + SA and APS + SA groups were significantly higher than those of the pair-fed groups (*P* < 0.05) (Fig. 3C). At 42 days of age, the abundance of *Firmicutes* in the CON + SA group was higher than that in the APS + SA group, whereas the abundance of *Bacteroidetes* in the APS + SA group was lower than that in the CON + SA group. In addition, the F/B ratio was lower for the APS + SA group than for the CON + SA group. The phyla *Tenericutes*, *Proteobacteria*, and *Synergistetes* were also common, accounting for 5.11%, 2.37%, and 0.45% of the total abundance, respectively (regardless of treatment).

The dominant families within the phylum *Bacteroidetes* were *Bacteroidaceae, Rikenellaceae*, *Tannerellaceae*, *Barnesiellaceae*, *Prevotellaceae*, and *Muribaculaceae*. The main families within the phylum *Firmicutes* were *Ruminococcaceae*, *Veillonellaceae*, *Lachnospiraceae*, *Lactobacillaceae*, and *Erysipelotrichaceae*. The dominant families belonging to the phylum *Proteobacteria* were *Burkholderiaceae*, *Xanthomonadaceae*, and *Sphingomonadaceae*. Other phyla (*Tenericutes, Synergistetes,* and *Fusobacteria*) present at very low relative abundance levels (Supplementary Table S2).

### The differentiated microbes of cecal microbiota in broilers

**Figure 4:**
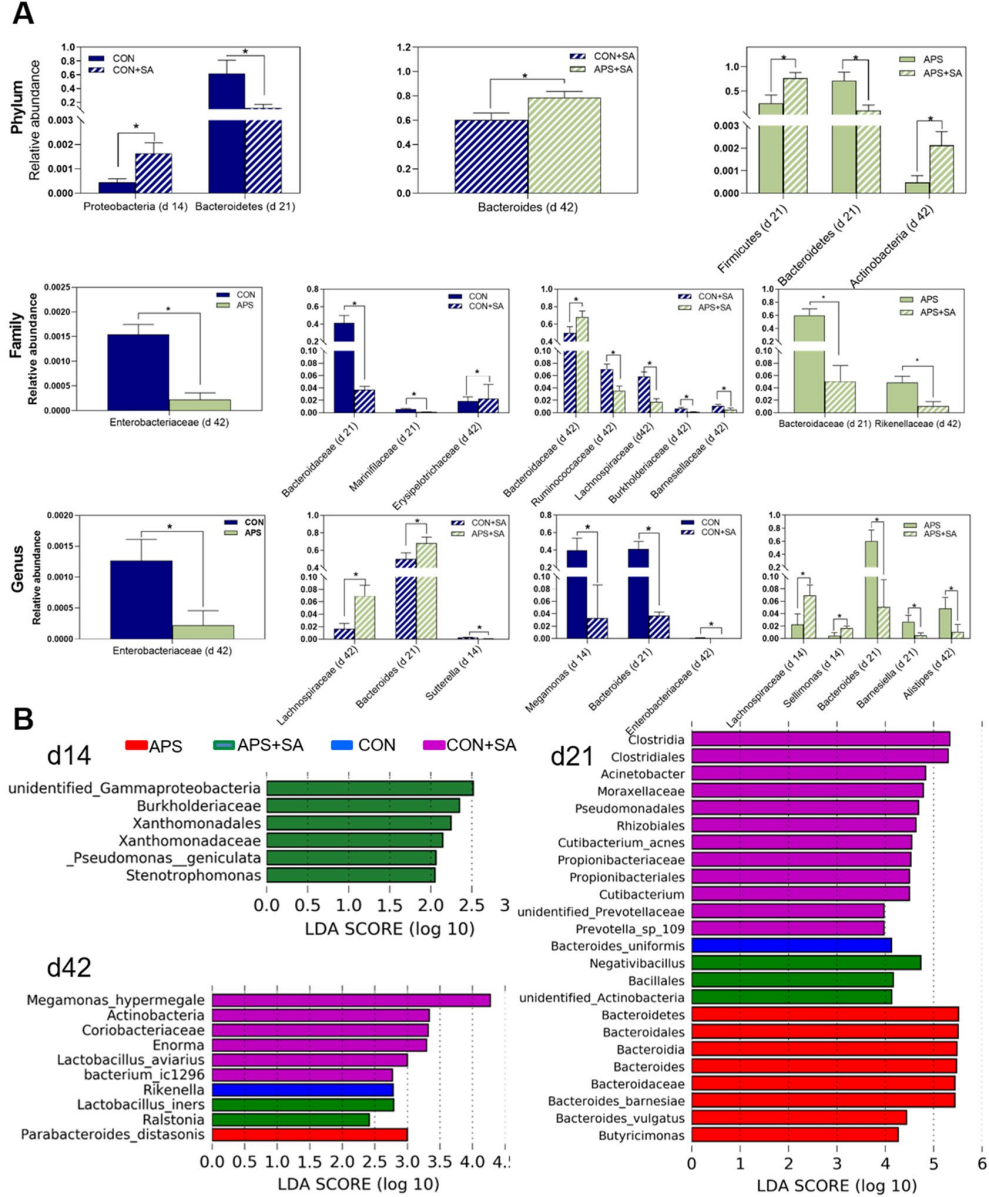
Cecal microbes with significant (*P* < 0.05) discrepancies in abundance among the different groups. (**A**) Differences in abundance at the phylum, family, and genus levels. The x-axis shows the microbes at the phylum, family, or genus level, and the numbers in parentheses indicate the ages of the broilers, (**B**) LefSe analysis of the cecal microbial communities in broilers from the CON, APS, CON + SA and APS + SA groups at 14, 21, and 42 days of age. The LDA histogram shown the microbial species with significant (*P* < 0.05) differences in different groups.

The discrepant microbes (biomarkers) are shown at the phylum, family, and genus levels in Fig. 4 and Supplementary Table S3. The structure and composition of the cecal microbiota were altered due to *S. ser. Enteritidis* infection and dietary supplementation with APS in broilers. Interestingly, *S. ser. Enteritidis* infection significantly (*P* < 0.05) decreased the abundance of the phylum *Bacteroidetes* while typically increased the abundance of *Firmicutes* (*P* < 0.05 on day 42) of microbiota in broilers regardless of APS supplemention or not (CON + SA group vs CON, APS + SA or APS group). In addition, the abundance of harmful *Proteobacteria* was notably higher in the CON + SA group than in the CON group (*P* = 0.03). Furthermore, *S. ser. Enteritidis*-infected broilers (CON + SA group) had lower abundance of *Bacteroidaceae* and *Bacteroides* than the CON or APS + SA group. However, compared to the control diet, the APS-supplemented broiler diet reduced the abundance of *Enterobacteriaceae*, a potential facultative anaerobic pathogen, at both the family and genus levels. Similarly, the abundance of the families *Erysipelotrichaceae*, *Ruminococcaceae*, *Lachnospiraceae*, *Burkholderiaceae*, and *Barnesiellaceae* was notably increased in the infected groups compared to the pair-fed groups. At the genus level, the abundance of *Lachnospiraceae* and *Sellimonas* was decreased, while that of *Barnesiella* and *Alistipes* was increased, in the APS group compared to the APS + SA group. In addition, the abundance of the genus *Sutterella* was increased in broilers in the CON + SA group compared to those in the APS + SA group. The genus *Megamonas* exhibited higher abundance in the CON group than in the CON + SA group.

Linear discriminant analysis (LDA) effect size (LefSe) analysis was also performed to determine the discrepant microbes among the four groups at different ages post infection (Fig. 4B, Supplementary Fig. S5). At 14 days of age, the abundance of certain facultative anaerobic microbes in the families *Burkholderiaceae*, *Xanthomonadaceae*, and *unidentified_Gammaproteobacteria*, and the genus *Stenotrophomonas* was significantly (*P* < 0.05) enhanced in the APS + SA group. At 21 days of age, the CON and APS groups exhibited enhanced abundance of the phylum *Bacteroidetes*, the family *Bacteroidaceae*, and the genera *Bacteroides* and *Butyricimonas*. On the other hand, the microbial communities of the *S. ser. Enteritidis*-challenged broilers (APS + SA and CON + SA groups) had elevated abundance of facultative anaerobic or potential pathogenic bacteria including *Clostridia*, *Acinetobacter*, *Moraxellaceae*, *Pseudomonadales*, and *Propionibacteriales*. At 42 days of age, the *Parabacteroides_distasonis* abundance was typically higher in the APS group than in the *S. ser. Enteritidis*-infected groups, and the *Rikenella* richness was greater in the CON group than in the *S. ser. Enteritidis*-infected groups. In addition, the APS + SA group had higher *Lactobacillus_iners* and *Ralstonia* abundance than the other groups, and the CON + SA group had greater *Megamonas*, *Actinobacteria*, *Coriobacteriaceae*, *Lactobacillus_aviarius*, and *Enorma* richness than the other groups. Thus, the alterations in the structure and composition of the cecal microbiota in *S. ser. Enteritidis*-challenged broilers exhibited time dependence.

### Comparison of metabolic pathway gene abundances and relative intestinal inflammatory cytokine levels

**Figure 5:**
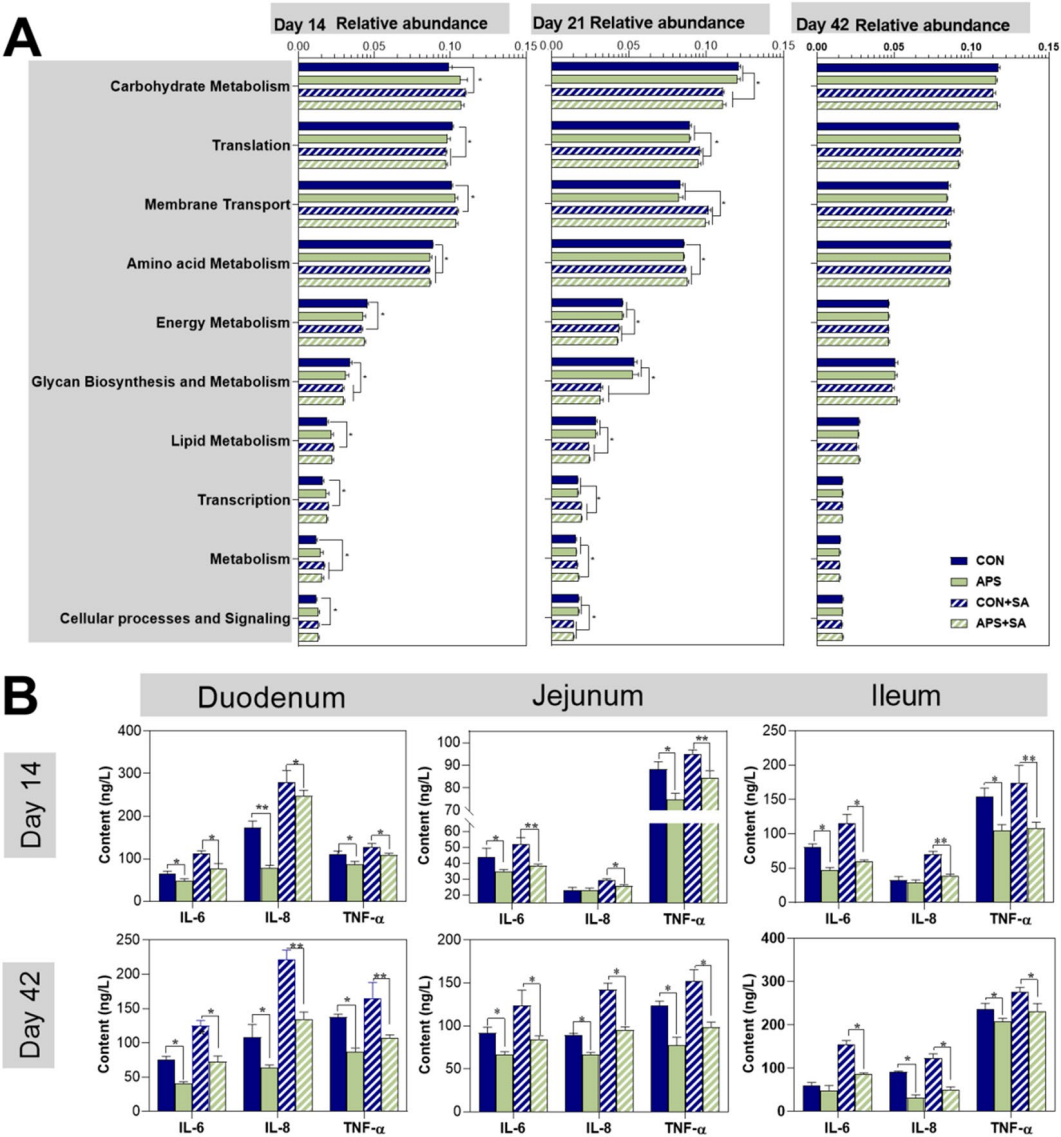
Effects of dietary APS supplementation on the functional genes abundance and inflammatory cytokines of *S. ser. Enteritidis*-infected broilers. (**A**) Abundance of certain functional genes related to metabolism or signaling pathways, (**B**) the expression of some inflammatory cytokines. CON, control pair-fed group; APS, APS-supplemented pair-fed group; CON + SA, control *S. ser. Enteritidis*-challenged group; APS + SA, APS-supplemented and *S. ser. Enteritidis*-challenged group. n = 6.

We predicted microbial metagenomes with 16S rRNA gene sequencing using phylogenetic investigation of communities by reconstruction of unobserved states (PICRUSt) ^17,18^ (the online procedure of Galax http://huttenhower.sph.harvard.edu/galaxy/) and found that the relative abundance of some genes related to metabolism and signaling pathways significantly (*P* < 0.05) varied with *S. ser. Enteritidis* infection or APS supplementation (Fig. 5A). To further study which metabolic genes changed with *S. ser. Enteritidis* infection and APS supplementation, 30 KEGG Orthology (KO) groups with a relative abundance above 0.5% were selected (Supplementary Table S4). In the early period after *S. ser. Enteritidis* infection (at 14 and 21 days of age), the abundance of most functional genes related to nutrient metabolism or relative signaling pathways were changed.

At 14 days of age, genes that regulated carbohydrate metabolism, lipid metabolism, membrane transport, transcription, and cellular processes and signaling were higher in the CON + SA group than in the CON group (*P* < 0.05), while similar richness was observed in the APS and APS + SA groups (*P* > 0.05). In contrast, the abundance of genes that modified amino acid metabolism, energy metabolism, and glycan biosynthesis and metabolism was lower in the CON + SA group than in the CON group (Fig. 5A). In addition, the bowel inflammatory cytokines IL-6, IL-8, and TNF-α were all enhanced in the CON + SA group compared to the other groups (Fig. 5B). The abundance of genes related to metabolism and signaling pathways was different at day 21 than at day 14. The CON and APS groups had higher abundance of genes involving carbohydrate metabolism, lipid metabolism, energy metabolism, glycan metabolism pathways, metabolism of cofactors and vitamins, and cellular processes and signaling than the CON + SA and APS + SA groups (*P* < 0.05). At 42 days of age, the abundance of all genes related to carbohydrate metabolism, energy metabolism, lipid metabolism, glycan biosynthesis and metabolism, and cellular processes and signaling did not significantly (*P* < 0.05) differ across different groups.

### Correlations between core gut bacteria related to broiler body weight (BW) and treatment-specific biomarker bacteria

**Figure 6:**
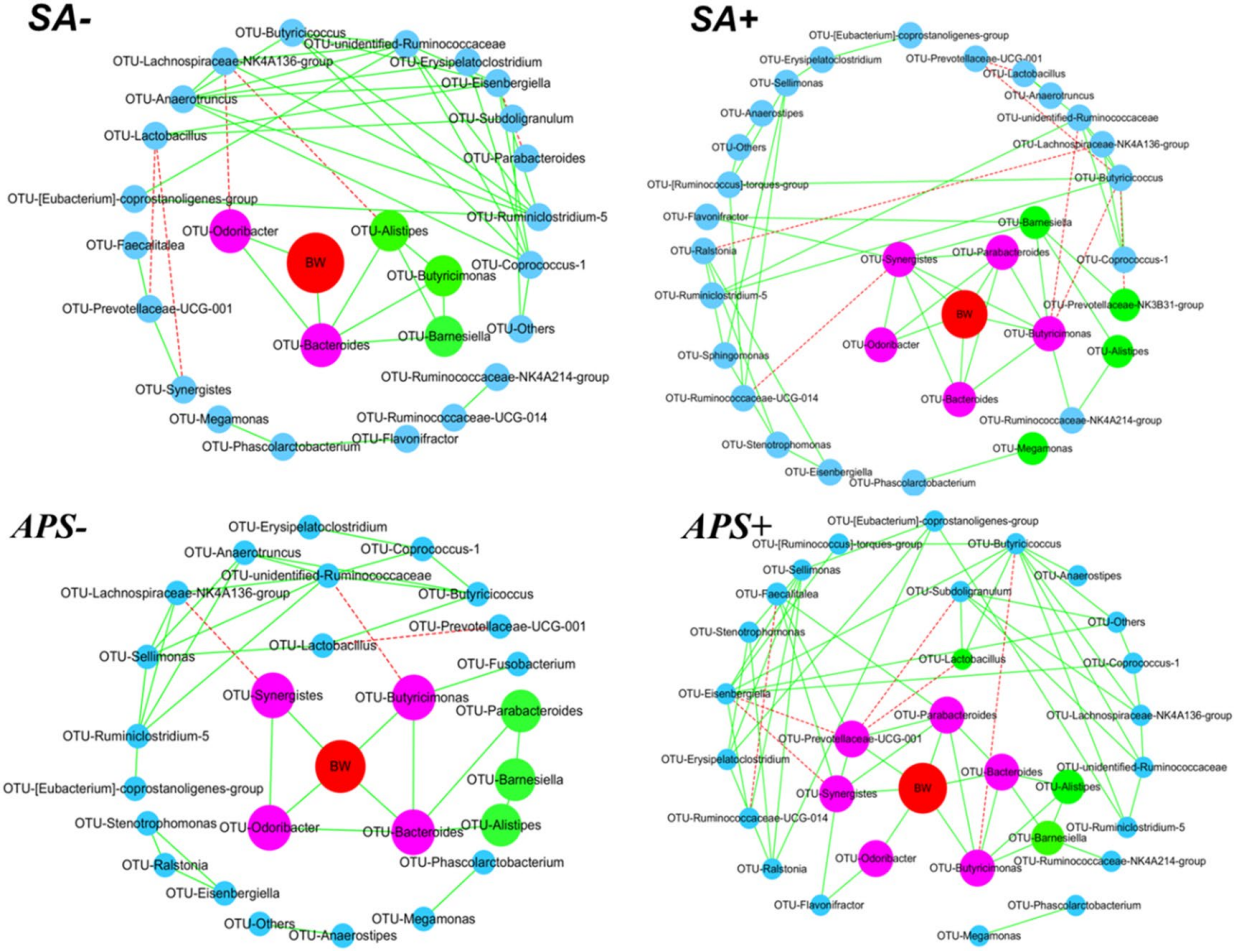
Co-occurrence network of cecal microbes and body weight (BW) in broilers challenged with *S. ser. Enteritidis* or dietary APS supplementation were made using cytoscape (3.8.2), https://cytoscape.org/. Here, we display the interactions among intestinal microbes at the genus level. The core bacteria (purple nodes) that are directly related to BW (red nodes) and the biomarker bacteria (green nodes) that interact (through synergy or mutual exclusion) with the core bacteria are shown. The abundance values of microbes connected with BW by a solid green line have a direct positive correlation with BW. Two microbes connected with a solid green line have a positive correlation, with a Spearman’s rank correlation coefficient higher than 0.60. Microbes connected with a dashed red have a negative relationship, with a Spearman’s rank correlation coefficient less than -0.60. *SA-*, pair-fed groups; *SA* + , *S. ser. Enteritidis*-challenged groups; APS-, no APS- supplemented groups; APS + : APS-supplemented groups.

To explore the correlations between intestinal microbes and phenotypic outcomes, a co-occurrence network was created with BW as the targeted factor, and the core microbes directly correlated with BW in broilers receiving different treatments were identified. As shown in Fig. 6, some different types of bacteria were directly related to BW in the different treatments groups. Under conditions of no *S. ser. Enteritidis* infection, two core microbes, *Odoribacter* and *Bacteroides*, were identified in the broiler cecal microbiota to have abundance values positively correlated with broiler BW. Moreover, the two bacteria presented synergistic interactions with some biomarker bacteria (bacteria that differed among groups), such as *Alistipes, Butyricimonas,* and *Barnesiella*, which jointly promoted the growth performance of broilers. When the broilers were infected with *S. ser. Enteritidis*, the core microbes directly correlated with BW included *Odoribacter*, *Bacteroides*, *Parabacteroides*, *Butyricimonas*, and *Synergistes*. In non-APS-supplemented broilers, the bacteria directly related to BW were *Odoribacter, Bacteroides*, *Butyricimonas*, and *Synergistes*. In broilers subjected to *S. ser. Enteritidis* challenge and dietary APS supplementation, the genera *Odoribacter*, *Bacteroides*, *Parabacteroides*, *Butyricimonas*, *Synergistes*, and *Prevotellaceae* exhibited direct correlations with broiler BW. Thus, dietary APS supplementation increased the abundance of core bacteria species directly related to BW. In addition, certain biomarker bacteria directly and positively cooperated with core microbes to construct a key subset of intestinal microbes that were directly related to BW, subsequently influencing broiler growth performance.

### Correlations between cecal microbes and healthy parameters

**Figure 7:**
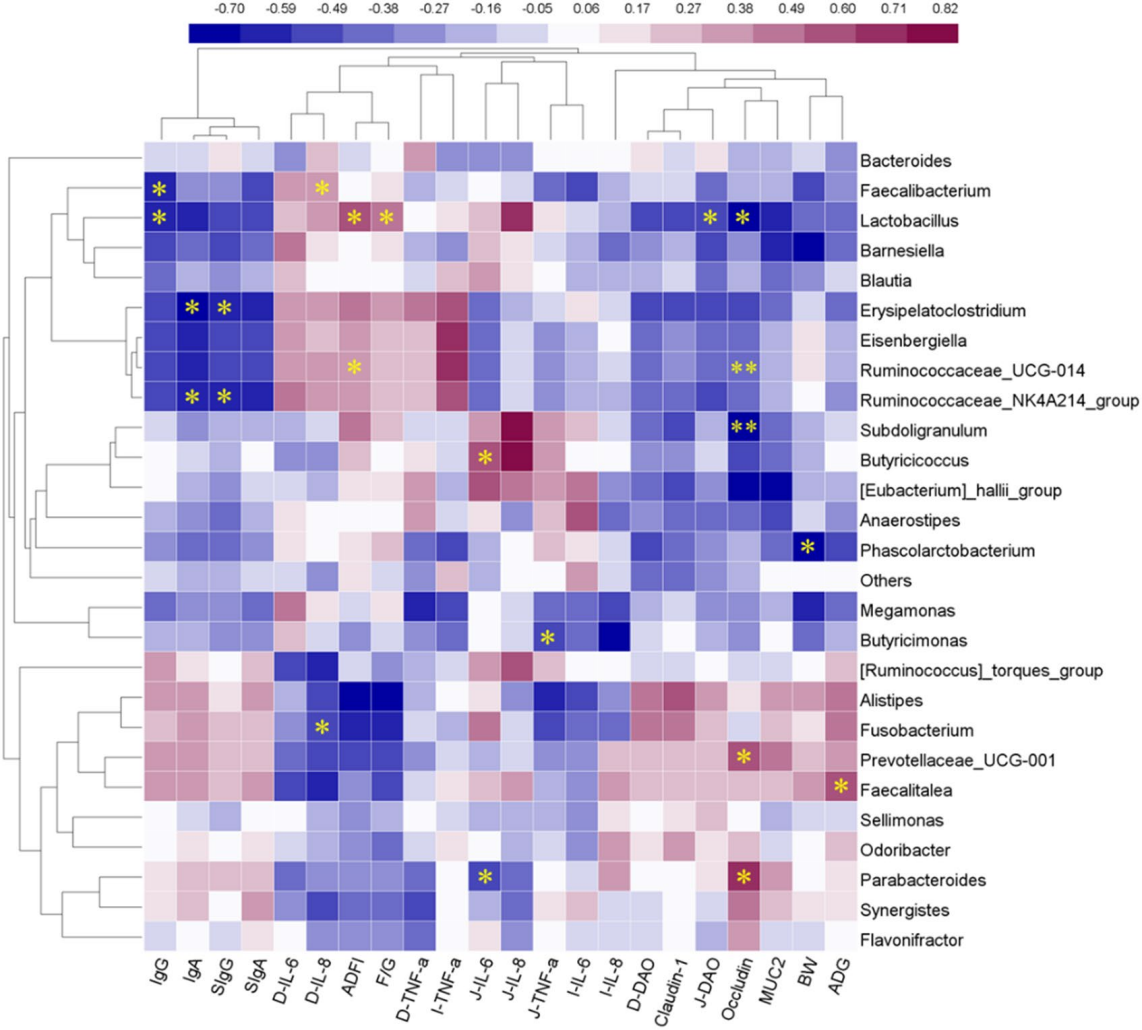
Correlations between significantly modified microbes (richness > 0.5%) and health parameters of broilers were analyzed by using Spearman’s correlation in SPSS Statistics 23.0 with the Bivariate correlation analysis and visualized with the heatmap (Heml 1.0.3.7, heatmap illustrator, http://hemi.biocuckoo.org/down.php). *P < 0.05, **P < 0.01 (Spearman’s correlation analysis). ADG, average daily gain; ADFI, average daily feed intake; F/G, ratio of ADFI to ADG; BW, body weight; IgG, Immunoglobulin G; IgA, Immunoglobulin A; sIgA, secretory IgA; sIgG, secretory IgG; D-IL-6, duodenal interleukin 6; D-IL-8, duodenal interleukin 8; D-TNF-α, duodenal tumor necrosis factor-α; J-IL-6, jejunal interleukin 6; J-IL-8, jejunal interleukin 8; J-TNF-α, jejunal tumor necrosis factor-α; I-IL-6, ileal interleukin 6; I-IL-8, ileal interleukin 8; I-TNF-α, ileal tumor necrosis factor-α; D-DAO, duodenal diamine oxidase; J-DAO, jejunal diamine oxidase.

A Spearman’s rank correlation analysis was performed to evaluate the potential links between alterations in cecal microbiota composition and relative growth and health parameters of broilers at 42 days of age (Fig. 7). The abundance values of the genera *Lactobacillus* and *Ruminococcaceae_UCG-014* were positively correlated with ADFI (*P* < 0.01). The abundance of the genus *Lactobacillus* was positively correlated with F/G, and that of the genus *Faecalitalea* was positively correlated with ADG. However, the abundance of the genus *Lactobacillus* was negatively correlated with IgA, J-DAO, and occludin expression. In addition, the abundance values of the genera *Lactobacillus* and *Faecalibacterium* were negatively correlated with IgG, and those of *Erysipelatoclostridium* and *Ruminococcaceae_NK4A214_group* were negatively correlated with IgA and sIgG. The abundance values of the genera *Faecalibacterium* and *Lactobacillus* were positively correlated with the duodenal inflammatory cytokine IL-8, and those of the genera *Parabacteroides* and *Butyricimonas* were negatively correlated with the jejunal inflammatory factors IL-6 and TNF-α, respectively. The genera *Prevotellaceae*, *Parabacteroides*, and *Butyricimonas* were positively correlated with increased expression of intestinal tight junction proteins (Occludin) and negatively correlated with intestinal inflammatory cytokine (J-IL-6, J-TNF-α) levels.

## Discussion

The current study investigated the effects of dietary APS supplementation on the performance and intestinal microbiota of *S. ser. Enteritidis*-challenged broilers. Our findings indicated that dietary APS supplementation improved the ADG while decreased ADFI and FCR compared with those of pair-fed broilers (CON + APS *vs.* CON; APS + SA *vs.* CON + SA), regardless of *S. ser. Enteritidis* infection. These observations are similar to the findings of Tong et al*.* (2004), who reported that broilers receiving diets supplemented with alfalfa extract containing APS had higher ADG and ADFI levels than those receiving control diet under *S. ser. Enteritidis*-infected conditions. Similarly, dietary APS supplementation has been found to improve the ADG, promote the intestinal morphology development, and increase the abundance of gut beneficial bacteria of piglets^19^. Those results suggested that the growth-promoting property of APS to animals.

In this study, the broilers fed APS-supplementated diet had the increased villus height, the ratio of V/C, and the decreased crypt depth in duodenum and jejunum, compared with no-supplemented broilers. Correspondingly, the increased intestinal DAO content and tight junction-related proteins (claudin-1, occludin, and MUC2) mRNA expression were also observed in APS-supplemented groups (APS and APS + SA groups) than those in the no APS-supplemented groups. Thus, the results verified the augmented intestinal barrier function and healthy status of broilers due to the dietary APS supplementation regardless of whether *S. ser. Enteritidis* infection. Similarly, Liu et al*.* (2018) observed that supplementation with *Achyranthes bidentata* polysaccharides in broiler’s diets improved the intestinal villus height and V/C ratios of broilers challenged with *Escherichia coli*.

We utilized inferred metagenomics by PICRUSt^17^ which can reflect the metabolic activities of the microbiota to investigate functional differences in the microbiota of broilers in order to determine the metabolic alterations caused by *S. ser. Enteritidis* infection or APS addition. The findings indicated that the genes of carbohydrate and lipid metabolism were more abundance at the first *S. ser. Enteritidis* infection of broilers, whereas those genes abundance significantly (*P* < 0.05) decreased at the second *S. ser. Enteritidis* administration in *S. ser. Enteritidis*-challenged group compared with the un-challenged group. The richness of glycan biosynthesis and metabolism genes were always lower in the *S. ser. Enteritidis*-challenged group than unchallenged-group, suggesting the decreased carbohydrate metabolism due to the *S. ser. Enteritidis* infection^17^. In addition, *S. ser. Enteritidis* infection in broilers resulted in severe bowel inflammation, as indicated by increased inflammatory cytokines of IL-6, IL-8, and TNF-α. This finding revealed that much more energy generated from carbohydrates and lipids metabolism was utilized to resist adverse stress and inflammation occurance induced by *S. ser. Enteritidis* infection rather than to promote the growth of broilers^20,21^, which might be the potential mechanism for decreased the growth performance^22^.

Regardless of *S. ser. Enteritidis* infection, the cecal microbiota was dominated by the phyla *Firmicutes* and *Bacteroidetes* in all groups, and the composition and structure of the gut microbial community exhibited a temporal shift in broilers with increasing age (at 14, 21, and 42 days of age). However, *S. ser. Enteritidis* infection delayed the changes of gut dominant bacteria from *Firmicute* to *Bacteroidetes* of broiers, while the dietary APS supplementation increased the *Bacteroidetes* abundance and decreased the ratio of *Firmicutes*/*Bacteroidetes* (F/B). The *Bacteroidetes* richness and the ratio of F/B were tightly related to the carbohydrates and lipid metabolism^22^, and the synthesis of bile acids and steroids^23^. In addition, *S. ser. Enteritidis* infection resulted in the enhanced abundance of facultative anaerobic bacteria or potential pathogens in cecal microbiota of broilers regarding the relative abundance of facultative anaerobic bacteria or potential pathogens in the phylum *Proteobacteria*; the families *Erysipelotrichaceae*, *Ruminococcaceae, Lachnospiraceae*, *Burkholderiacea*e, and *Barnesiellaceae*; and the genera *Lachnospiraceae* and *Sutterella*, which directly destroyed the intestinal microbiota ecosystem and induced bowel inflammation^22,24^. Conversely, dietary APS supplementation reduced the proliferation of pathogens in intestine and enhanced the abundance of certain beneficial bacteria, including *Bacteroidetes, Parabacteroides distasonis*, and *Lactobacillus_iners.* These results were consistent with the greater expression of the inflammatory cytokines IL-6, IL-8, and TNF-α observed in the gut mucosa in the *S. ser. Enteritidis*-infected groups. Therefore, the increased abundance of potential pathogens in *S. ser. Enteritidis*-infected broilers was considered the causal mechanism for the observed gut inflammation and deteriorated physiological conditions^13,25^.

The co-occurrence analysis between the cecal microbiota and BW revealed that both *S. ser. Enteritidis* infection and dietary APS supplementation increased the abundance of core microbe taxa directly related to BW, including *Odoribacter*, *Bacteroides*, *Parabacteroides*, *Butyricimonas*, *Synergistetes*, and *Prevotellaceae*. Interestingly, these genera all belong to the phylum *Bacteroidetes* and have diverse physiological functions, including maintenance of gut integrity and improvement of immunity. Moreover, *Odoribacter* and *Bacteroides* appeared across all treatments, regardless of *S. ser. Enteritidis* infection and APS supplementation. *Parabacteroides distasonis* was considered as a beneficial bacterium in the bowel that modulated host metabolism and alleviated metabolic dysfunction by producing succinate and secondary bile acids^26^. Similarly, *Butyricimonas* bacteria can improve intestinal barrier functions by producing short-chain fatty acids, β-galactosidase, N-acetyl-β-glucosaminidase, indole, leucyl glycine and pyroglutamic acid arylamidase from the fermentation of polysaccharides such as APS^27,28^. Thus, the genera *Bacteroides*, *Parabacteroides*, *Butyricimonas,* and *Prevotellaceae* are core microbe taxa directly related to BW, which combined with their syntrophic microbes including *Alistipes*, *Barnesiella*, and *Lactobacillus* formed the hub in co-occurrence networks linking microbiome structure to host body weight of broilers. The correlation analyses indicated that bacteria related to the production of short-chain fatty acids, including *Lactobacillus, Faecalibacterium, Faecalitalea,* and *Ruminococcaceae_UCG-014*, exerted significant (*P* < 0.05) effects on growth performance. Furthermore, bacteria related to carbohydrate and lipid metabolism, such as *Odoribacter*, *Bacteroides*, *Parabacteroides*, *Butyricimonas*, *Synergistetes*, and *Prevotellaceae*, showed direct correlations with BW under conditions of dietary APS supplementation and *S. ser. Enteritidis* infection^29^.

Taken together, regardless of *S. ser. Enteritidis* infection, dietary APS supplementation modulated the configuration of gut microbiota of *S. ser. Enteritidis*-challenged broilers with the decreased F/B ratio, improved abundance of beneficial bacteria and the increased amounts of body weight-related core bacteria, which contributed to the improvement of intestinal morphology, mucosal barrier function, and growth performance^30^. The identified core microbes and their syntrophic partners that tightly correlated to BW might be a new modulatory target for developing a dietary strategy to alleviate the *S. ser. Enteritidis* infection adverse and improve the production performance of broilers.

## Conclusions

Taken together, regardless of whether *S. ser. Enteritidis* infection was present, dietary supplementation with APS improved the growth performance and systematic health of broilers. Feeding APS-supplemented diet to broilers decreased the F/B ratio and enhanced the richness of beneficial bacteria mainly involving *Bacteroidetes*, *Bacteroidetes*, *Barnesiella*, *Alistipes*, *Parabacteroides*, *Butyricimonas*, and *Prevotellaceae*, whereas *S. ser. Enteritidis* infection resulted in the increased abundance of pathogens referring to the *Protecbacteria*, *Actinobacteria*, *Erysipetotrichaceae*, and *bacterium_ic1296* in cercal microbiota of broilers. In addition, the increased beneficial bacteria due to the APS supplementation exhibited the positive correlation to the production and healthy parameters. The *Bacteroides* and *Odoribacter* were identified as the two core microbes across all treatments and combined with their syntrophic microbes formed the hub in co-occurrence networks linking microbiome structure to host body weight of broilers. Thus, regardless of *S. ser. Enteritidis* infection, dietary supplementation with APS manipulated the configuration of gut microbiota, which contributed to the improved the performance and systemtic health of broilers. The identified core microbes and their syntrophic partners are potential primary targets for dietary strategy to enhance growth performance and food safety in the poultry industry.

## Materials and methods

### Ethics statement

This study was conducted in accordance with the animal care and use protocol approved by the Ethics Committee of Animal Experiment of Animal Nutrition Institute of Shandong Agricultural University (approved No.: SD2019-0318), and the protocols were in accordance with the Chinese legislation on animal experimentation, which comply with the ARRIVE guidelines 2.0^31^. The experiment was carried out at the animal experiment station of Shandong Agricultural University (Taian, China).

### Alfalfa polysaccharide preparation

APS was prepared according to a previously described extraction and purification procedure, and the composition and molecular characteristics of APS have been verified^15,17^. Briefly, the oven-dried alfalfa sample was immersed with double-distilled water in the ratio of 1: 10 (alfalfa: distilled water), boiled and kept simmering for 4 h, condensing the liquid to a quarter of its original volume. Subsequently, the liquid was filtered through two layers of nylon mesh (0.2-cm mesh), after cooling, mixed with the 5% trichloroacetic acid (TCA) (v:v, 1:2 = filter liquid:TCA), and stay for 2 h to precipitate protein in the filtrate. Then the liquid fraction was centrifuged at 3000 × g for 10 min, and the supernatants was transferred to another container and added 4 times of absolute ethyl alcohol (v/v). The mixture was kept at 4 °C for 12 h then centrifuged at 3000 × g for 10 min to precipitate crude polysaccharide. The crude polysaccharide pellet was subsequently re-dissolved in deionized H_2_O (d-H_2_O) and dialyzed using a biological semipermeable membrane (Molar mass > 8000 D, Beijing Solarbio Science and Technology Co., Ltd., Beijing, China) against d-H_2_O (10 times the sample volume) at 4 °C for 48 h, with changing the dH_2_O every 12 h. The sediment was collected and lyophilized using a vacuum dryer (Biosafer-10A, Biosafer, Nanjing, China) to a constant weight, which was considered as purified APS. The molecular characteristics involving the compositions, glycosidic linkage between monsaccharide, molecular mass of APS have been clarified in our previous study^15^. In brief, APS was composed of fucose, arabinose, galactose, glucose, xylose, mannose, galactose, galacturonic acid (GalA), and glucuronic acid (GlcA) with a molar ratio of 2.6:8.0:4.7:21.3:3.2:1.0:74.2:14.9. The weight-average molecular weight (Mw) was 3.30 × 10^6^ g/mol. The intrachain glycosidic linkage mainly consists of 1,5-Araf, galactose (T-D-Glc), glucose (T-D-Gal), 1,4-Gal-Ac, 1,4-Glc, 1,6-Gal, and 1,3,4-GalA, with molar proportions of 10.30%, 4.02%, 10.28%, 52.29%, 17.02%, 3.52%, and 2.57%, respectively.

### Experimental design and animals

Two hundred and forty 1-day-old vaccinated (against Marek’s disease and infectious bronchitis) Arbor Acres broiler chicks (mixed sex) were obtained from a local commercial hatchery in China. The broilers were randomly allocated into 24 pens of 4 treatments (10 birds per pen and 6 pens per treatment). The experiment was conducted with a two-factor factorial design, and the pens were considered replicate units. The 4 treatment groups were as follows: (1) a basal diet-fed group (the CON group), (2) a basal diet-fed group challenged with 3 mL of *S. ser. Enteritidis* by oral gavage at 11 and 18 days of age (the CON + SA group), (3) an APS-supplemented basal diet-fed group (dose: 500 mg/kg diet; the APS group), and (4) an APS-supplemented basal diet-fed group challenged with *S. ser. Enteritidis* (the APS + SA group). Of them, the CON group VS. APS group, the CON + SA group VS. the APS + SA group, were separated taken as the paired-fed groups in data statistics.

The basal diet (Table 3) was formulated to meet the nutritional requirements recommended by the feeding standards for broilers in China (NY/T 33–2004). All diets were prepared in a single batch and stored in a cool warehouse. The APS was first combined with a premix that was subsequently mixed with other ingredients and then stored in covered containers^31^. The birds were maintained in an environmentally controlled house. The temperature was maintained at 32 °C from day 1 to 7, gradually decreased to 20 °C at a rate of 3 °C per week, and then maintained at 20 °C until the end of the trial. The light cycle was 24 h from days 1 to 3, 18 h from days 4 to 20, 21 h from days 21 to 35, and 23 h from days 36 to 42 of the experiment^32^. The birds were fed ad libitum and had free access to water through nipple drinkers for the entire duration of the experiment .

**Table 3:** Compositions and nutrient levels of the basal diets.

Item	Diet composition in each of two phases	
	Day 1–21	Day 22–42
**Ingredients, g/kg**		
Corn	510	544
Soybean meal	323	291
Wheat bran	80	80
Soybean oil	45	45
DL-Methionine	2	1
Calcium carbonate	15	15
Calcium phosphate	16	15
Sodium chloride	4	4
Premix^1^	5	5
Total	1,000	1,000
**Nutrient levels**^**2**^**, g/kg**		
Metabolic energy (ME), MJ/kg DM	12.74	12.89
Crude protein	203	191
Lysine	9.9	9.7
Methionine	5.2	4.9
Calcium	10	9
Phosphorus	4.5	3.5

^1^Supplied per kilogram of complete diet: 11,025 IU vitamin A, 2,203 IU vitamin D_3_, 80 IU vitamin E, 4.4 mg vitamin K_3_, 4.4 mg thiamine, 11 mg riboflavin, 35 mg d-pantothenic acid, 59.5 mg niacin, 330 mg choline, 0.9 mg folic acid, 0.5 mg biotin, 55 μg vitamin B_12_, 40 mg Mn as manganese sulfate, 130 mg Fe as ferrous sulfate, 130 mg Zn as zinc sulfate, 15 mg Cu as copper sulfate, 0.35 mg I as calcium iodide, and 0.3 mg Se as sodium selenite.

^2^All items of nutrient level except ME were measured values (n = 6).

ADG and ADFI were determined weekly by determining the BW of the birds and their feed consumption per cage (sum of feed offered—feed leftover at weighing time). The FCR was calculated as the ADFI divided by the corresponding ADG. The ADG, ADFI, and FCR were determined separately for the starter period (days 1 to 21), grower period (days 22 to 42), and entire feeding period (days 1 to 42)^32,33^.

### *S. ser. Enteritidis* infection procedure

Freeze-dried cultures of a *S. ser. Enteritidis* enteritidis serotype obtained from the China Veterinary Culture Collection Center (Beijing, China, Catalog no. CVCC3377, http://61.153.187.228:8081/web/shopcart/ProductsSetting_Virus_Detail.aspx?id=CVCC3377) were rehydrated in 10 mL of sterile tryptone soy broth (CM201, Land Bridge Technology, Ltd., Beijing, China). Drops of the suspension were plated 2 times successively on xylose lysine deoxycholate medium (Land Bridge Technology, Ltd.,) for 24 h at 37 °C. Then, 30 mL of preculture was prepared by picking a single colony into sterile pre-warmed tryptone soy broth and incubating the mixture at 37 °C with orbital shaking for 24 h. Subsequently, 10 mL of *S. ser. Enteritidis* preculture was transferred into 300 mL of sterile tryptone soy broth, and the mixture was incubated in an orbital shaker at 37 °C for 16 h. The number of colony-forming units (CFU) was determined with a tenfold dilution series in sterile buffered 0.9% peptone water with a pH of 7.2. The stock culture was adjusted to 1 × 10^7^ CFU by centrifuging at 3,000 rpm and 4 °C for 10 min^34^. The *S. ser. Enteritidis* solution was freshly prepared before oral gavage. A pilot trial was conducted to ascertain the appropriate dose administrated to broilers^34^(Supplement 1. pilot trial).

### Cecal sample collection and microbiota determination

Six broilers (1 bird per pen) were randomly selected from each treatment in the morning on days 14, 21 and 42 of the experiment after 12 h of fasting and were weighed. The birds were sacrificed by cervical dislocation, and the abdominal cavity of each bird was immediately opened. Fresh chyme samples were collected by grabbing them directly from the ceca of 6 healthy broilers in each treatment group on days 14, 21, and 42 of the experiment. Those broilers appeared to be in an energetic condition with normal intake, neat feathers, and no diarrhea, as well as have no inflammation in the intestines and other organs after dissection. The samples were stored in 5-mL cryogenic vials and were immediately flash-frozen in liquid nitrogen until analysis.

DNA from the caecal content samples were extracted using QIAamp DNA Stool Mini Kits (Qiagen Inc., Hilden, Germany) according to the manufacturer’s instructions. DNA concentration and quality were assessed using a Qubit 2.0 fluorometer (Invitrogen, ThermoFisher scientific, MA) and gel electrophoresis^35,36^. The V3-V4 hyervariable region of the 16S rRNA gene was amplified with universal primers 341F (CCTACGGGNGGCWGCAG) and 805R (GACTACHVGGGTATCTAATCC) as described by Pourabedin, M. et al. (2015) and Pandit, R. J. et al.^7,36^. Amplicon libraries were sequenced on Illumina NovaSeq 6000 platform (Novogene, Beijing, China) for paired-end reads of 500 bp (PE250). The raw paired-end reads were assembled into longer sequences and quantitatively filtered by Qiime (version 1.9.1, http://qiime.org/scripts/split_libraries_fastq.html) to remove the low-quality reads (Tags intercepting: Removed RAW Tags from a continuous low quality value (quality threshold <  = 19) truncation of the first low quality base site when the number of bases reaches the set length (the default length is 3); Tags length filtering: Tags data set obtained after Tags are cut off, and Tags whose continuous high-quality base length is less than 75% of the length of Tags are further filtered out.)The high-quality sequences were clustered into OTUs with a 97% similarity using Uparse (version 7.0.1001, http://www.drive5.com/uparse/ (http://www.drive5.com/uparse/) in Qiime (version 1.9), and the chimeras were removed using Uresearch (http://drive5.com/usearch/manual/uparse_cmds.html). Taxonomy was assigned to OTUs using the RDP (Ribosomal Database Project) classifier against the SILVA 132(http://www.arb-silva.de/) SSUrRNA gene database (Release1282), with a confidence threshold of 80% ^37^ . All the raw data involved in the present study were deposited in NCBI database with the BioProject PRJNA579468.

### Determination of small intestinal morphology

To elucidate the effects of APS supplemented in diet on digestive tract development, the intestine slices were made to estimate the morphology variation by microscopic inspection as the method described by Zhang et al. (2019). Intestinal morphological measurements were conducted on 21- and 42- d-old broilers (6 broilers for each age) included villus height, crypt depth, and the ratio villus to crypt (V/C). Two 3-cm segments of mid-duodenum and mid-jejunum were removed with scalpel and rinsed with salt solution (9 g/L, w/v), and fixed with 100 g/L (w/v) formaldehyde-phosphate buffer, dehydrated, and embedded in paraffin wax. Serial Sects. (5-µm thickness) were obtained using a microtome and stained with hematoxylin and eosin (H&E). Ten intact and well-oriented villi and their associated crypts from each segment were measured with a light microscope (BX-51, Olympus, Tokyo, Japan) equipped with Image-Pro Plus software (version 6.0, Motic Images software, Motic China Group Co., Ltd., Xiamen, China). Villus height was measured from villi tip to villus-crypt junction, and crypt depth was defined as depth of invagination between adjacent villi^38^. The value of the villus height divided by the crypt depth was defined as the villus-to-crypt ratio (V:C).

### Determination of diamine oxidase (DAO), tight junction proteins, and inflammatory cytokines

After the 6 birds were sacrificed and three 3-cm segments were successively taken from the proximal duodenum, jejunum, and ileum for analysis of mucosal DAO, tight junction protein (occludin-1, occludin, and MUC2) mRNA expression, and inflammatory cytokine (IL-6, IL-8, and TNF-α). The relative mRNA expression of tight junction proteins such as claudin-1, occludin, and MUC2 in the jejunum was determined to investigate intestinal barrier integrity. The detailed procedure was list in Supplement 2, Table S1. (Determination of intestinal tight junction protein expression). The DAO concentrations in the duodenal and jejunal mucosa and the IL-6, IL-8, and TNF-α concentrations in the duodenum, jejunum, and ileum were determined using broiler ELISA kits with standard curves according to the manufacturer’s instructions (Shanghai Yili biotechnology co., Ltd., Shanghai, China). All procedures were performed with 3 repetitions.

### Determination of serum IgA/G and intestinal mucosal SIgA/G

Before sacrificing, blood samples (2.0 mL) were taken from the wing veins of 42-day-old birds into 2 tubes. One of the blood samples of each bird was incubated in a water bath at 37 °C for 2 h and subsequently centrifuged at 1500 × g for 10 min at room temperature; the separated serum was stored in a 1.5-mL Eppendorf tube at − 80 °C for further analysis of IgA and IgG levels. The collected duodenum mucosa (1 g) was mixed with an equal wight/volume of phosphate-buffered saline (PBS) (pH 7.14) and centrifuged at 1000 × g for 15 min. The supernatant was collected forl SIgA and SIgG determination. The content of serum IgA/G and duodenum SIgA/G were detected by broiler enzyme-linked immunosorbent assay (ELISA) kits (Nanjing Jiancheng Bioengineering Institute, Nanjing, China).

### Statistical analysis

Data shown are means ± standard error of the mean (SEM). Data were analyzed by one-way ANOVA followed by Dunn’s multiple comparisons (Prism 8.0) if the data were in Gaussian distribution and had equal variance or analyzed by the Kruskal–Wallis test followed by Dunn’s multiple comparisons if the data were not normally distributed. Differences with *P* < 0.05 were considered significant. Linear discriminant analysis (LDA) effect size analysis of ruminal microbiota changes was conducted using the online procedure of Galax (http://huttenhower.sph.harvard.edu/galaxy/--LEfSe). Co-occurrence network involved differentiated cecal microbes and body weight (BW) of broilers in different treatments were made using cytoscape (3.8.2 https://cytoscape.org/) for ascertaining the core microbes that directly correlated with BW. Correlations between significantly modified microbes (richness > 0.5%) and health parameters of broilers were analyzed by using Spearman’s correlation in SPSS Statistics 23.0 with the Bivariate correlation analysis and visualized with the heatmap (Heml 1.0.3.7, heatmap illustrator, http://hemi.biocuckoo.org/down.php).

## Supplementary Information

Supplementary Information.

## References

[1] Humphrey T (2004). Salmonella, stress responses and food safety. Nat. Rev. Microbiol..

[2] Hardie KM, Guerin MT, Ellis A, Leclair D (2019). Associations of processing level variables with salmonella prevalence and concentration on broiler chicken carcasses and parts in Canada. Prev. Vet. Med..

[3] Jeong J, Chon J, Kim H, Song K, Seo K (2018). Risk assessment for salmonellosis in chicken in south korea: the effect of concentration in chicken at retail. Korean J. Food Sci. Anim. Resour..

[4] Yang Y (2018). Salmonella excludes salmonella in poultry: confirming an old paradigm using conventional and barcode-tagging approaches. Front. Veterinary Sci..

[5] Zhen W (2018). Effect of dietary bacillus coagulans supplementation on growth performance and immune responses of broiler chickens challenged by salmonella enteritidis. Poult. Sci..

[6] Cheng YF (2019). Dietary mannan oligosaccharide ameliorates cyclic heat stress-induced damages on intestinal oxidative status and barrier integrity of broilers. Poult. Sci..

[7] Pourabedin M, Guan L, Zhao X (2015). Xylo-oligosaccharides and virginiamycin differentially modulate gut microbial composition in chickens. Microbiome..

[8] Wang L (2018). Lactobacillus plantarum restores intestinal permeability disrupted by salmonella infection in newly-hatched chicks. Sci. Rep..

[9] Chen HL (2003). Effects of Chinese herbal polysaccharides on the immunity and growth performance of young broilers. Poult. Sci..

[10] Islam MM, Yang C (2017). Efficacy of mealworm and super mealworm larvae probiotics as an alternative to antibiotics challenged orally with salmonella and e.coli infection in broiler chicks. Poult. Sci..

[11] Li J (2017). The effects of different enrofloxacin dosages on clinical efficacy and resistance development in chickens experimentally infected with salmonella typhimurium. Sci. Rep..

[12] Neveling DP (2017). Safety assessment of antibiotic and probiotic feed additives for gallus gallus domesticus. Sci. Rep..

[13] Parsons BN (2014). Dietary supplementation with soluble plantain non-starch polysaccharides inhibits intestinal invasion of salmonella typhimurium in the chicken. PLoS ONE.

[14] Kanwal S (2018). A polysaccharide isolated from dictyophora indusiata promotes recovery from antibiotic-driven intestinal dysbiosis and improves gut epithelial barrier function in a mouse model. Nutrients.

[15] Zhang C (2019). Extract methods, molecular characteristics, and bioactivities of polysaccharide from alfalfa (*Medicago Sativa* L.). Nutrient..

[16] Wang L (2019). Alfalfa polysaccharide prevents ho-induced oxidative damage in mefs by activating mapk/Nrf2 signaling pathways and suppressing Nf-Κb signaling pathways. Sci. Rep..

[17] Langille MG (2013). Predictive functional profiling of microbial communities using 16s Rrna marker gene sequences. Nat. Biotechnol..

[18] Fadrosh DW (2014). An improved dual-indexing approach for multiplexed 16s Rrna gene sequencing on the illumina miseq platform. Microbiome..

[19] Zhang CY (2019). Effects of dietary supplementation of alfalfa polysaccharides on growth performance, small intestinal enzyme activities, morphology, and large intestinal selected microbiota of piglets. Livest. Sci..

[20] Yan H (2017). Intake of total saponins and polysaccharides from polygonatum kingianum affects the gut microbiota in diabetic rats. Phytomedicine.

[21] Li Y (2018). Transgenerational effects of paternal dietary astragalus polysaccharides on spleen immunity of broilers. Int. J. Biol. Macromol..

[22] Pan S (2017). Effect of high dietary manganese on the immune responses of broilers following oral salmonella typhimurium inoculation. Biol. Trace Elem. Res..

[23] Mariat D (2009). The firmicutes/bacteroidetes ratio of the human microbiota changes with age. Bmc Microbiol..

[24] Khalili H (2018). The role of diet in the aetiopathogenesis of inflammatory bowel disease. Nat. Rev. Gastro. Hepat..

[25] El-Deek AA (2020). Alternative feed ingredients in the finisher diets for sustainable broiler production. Sci. Rep..

[26] Wang K (2019). Parabacteroides distasonis alleviates obesity and metabolic dysfunctions via production of succinate and secondary bile acids. Cell Rep..

[27] Ulger Toprak N, Bozan T, Birkan Y, Isbir S, Soyletir G (2015). Butyricimonas virosa: the first clinical case of bacteraemia. New Microbes New Infect..

[28] Cooper C, Moore SC, Moore RJ, Chandry PS, Fegan N (2018). Salmonella enterica subsp. salamae serovar sofia, a prevalent serovar in Australian broiler chickens, is also capable of transient colonisation in layers. Brit. Poultry Sci..

[29] Kolodziejczyk, A. A., Zheng, D. & Elinav, E. Diet–Microbiota Interactions and Personalized Nutrition. *Nat. Rev. Microbiol.* 1–12 (2019). 10.1038/s41579-019-0256-8 31541197

[30] Percie Du Sert N (2020). Reporting animal research: explanation and elaboration for the arrive guidelines 20. Plos Biol..

[31] Lahouar L (2012). Effect of dietary fibre of barley variety ‘rihane’on azoxymethane-induced aberrant crypt foci development and on colonic microbiota diversity in rats. Brit. J. Nutr..

[32] Zhang GG, Yang ZB, Wang Y, Yang WR (2013). Effects of astragalus membranaceus root processed to different particle sizes on growth performance, antioxidant status, and serum metabolites of broiler chickens1. Poult. Sci..

[33] Zhang GG, Yang ZB, Zhang QQ, Yang WR, Jiang SZ (2012). A multienzyme preparation enhances the utilization of nutrients and energy from pure corn and wheat diets in broilers. J. Appl. Poultry Res..

[34] Basit, M. A. et al. Comparative Efficacy of Selected Phytobiotics with Halquinol and Tetracycline On Gut Morphology, Ileal Digestibility, Cecal Microbiota Composition and Growth Performance in Broiler Chickens. *Animals*, 2020. 10.3390/ani10112150 PMC7699210 33227911

[35] Xiong W (2018). Antibiotic-mediated changes in the fecal microbiome of broiler chickens define the incidence of antibiotic resistance genes. Microbiome..

[36] Pandit RJ (2018). Microbial diversity and community composition of caecal microbiota in commercial and indigenous indian chickens determined using 16s rdna amplicon sequencing. Microbiome..

[37] Pruesse E (2007). Silva: a comprehensive online resource for quality checked and aligned ribosomal rna sequence data compatible with arb. Nucleic Acids Res..

[38] Xie YH (2018). Effects of dietary supplementation of enterococcus faecium on growth performance, intestinal morphology, and selected microbial populations of piglets. Livest. Sci..

