# Supplementary material for: The modulatory effects of alfalfa polysaccharide on intestinal microbiota and systemic health of Salmonella serotype (ser.) Enteritidis-challenged broilers
Source: Sci Rep. 2021 May 25;11:10910. doi: 10.1038/s41598-021-90060-6 (PMC8149654; doi:10.1038/s41598-021-90060-6)
Supplement: Supplementary file 1 — Supplementary Information. [file 41598_2021_90060_MOESM1_ESM.docx]

**Supplementary materials**

**The Modulatory Effects of Alfalfa Polysaccharide on Intestinal Microbiota and Systemic health of *Salmonella serotype (ser.) Enteritidis*-challenged broilers**

Zemin Li ^1,†^, ChongYu Zhang^1,†^, Bo Li ^1,†^, Shimin Zhang ^1,†^ , Fawaz G Haj^2^, Guiguo Zhang ^1,2^*, Yunkyoung Lee ^3,^*

^1^ Department of Animal Nutrition, Shandong Agricultural University, 61 Daizong Street, Taian City 271018, China;

^2^ Department of Nutrition, University of California Davis, One Shields Ave, Davis, CA 95616, USA;

^3^ Department of Food Science and Nutrition, and Interdisciplinary Graduate Program in Advanced Convergence Technology & Science, Jeju National University, Jeju 63243, South Korea; lyk1230@jejunu.ac.kr

***** Correspondence: [zhanggg@sdau.edu.cn](mailto:zhanggg@sdau.edu.cn) (G. Z.);  [lyk1230@jejunu.ac.kr](mailto:lyk1230@jejunu.ac.kr) (Y.L.)

Tel.: +86-5388-241544 (ext. 8327), +82-64-754-3555

† These authors contributed equally to this study and share the first authorship.

**Supplement 1 pilot trial**

*To explore the appropriate dose of Salmonella serotype (ser.) Enteritidis infection to broilers*

Prior to the feeding experiment, a pilot trial was carried out to determine the appropriate dose of *Salmonella serotype (ser.) Enteritidis* solution to give to broilers by oral gavage and to observe *S. ser. Enteritidis* translocation and colonization in tissue. Four doses (0 (control), 2 mL, 3 mL, and 4 mL/bird; 6 birds/group) of *S. ser. Enteritidis* solution (concentration: 1.0×10^7^ CFU/mL) were given to 24 broilers at 8 days of age by oral gavage for 3 consecutive days. The birds were then sacrificed at 15 days of age, and the livers were collected and cultured on *Salmonella* *Shigella* (SS) agar substrate to observe *S. ser. Enteritidis* proliferation in the liver (CFU/g) on the 7^th^ day post infection (Figure S1, B). The birds were fasted for 10 h before being challenged with *S. ser. Enteritidis* solution. The infection trial chickens were treated for 3 consecutive days by oral gavage with 3 mL of *S. ser. Enteritidis* *enteritidis* solution at 1×10^7^ CFU/mL, and the infection protocol was repeated at 7-day intervals (on days 11 and 18) (Figure S1, A); the pair-fed chickens were inoculated with sterilized sterile tryptone soy broth without *S. ser. Enteritidis* on the same days (Kanwal et al., 2018). The growth performance, physiological conditions, intestinal development, and gut microbiota were analyzed. The sample collection and *S. ser. Enteritidis* infection times are shown in Figure S1.


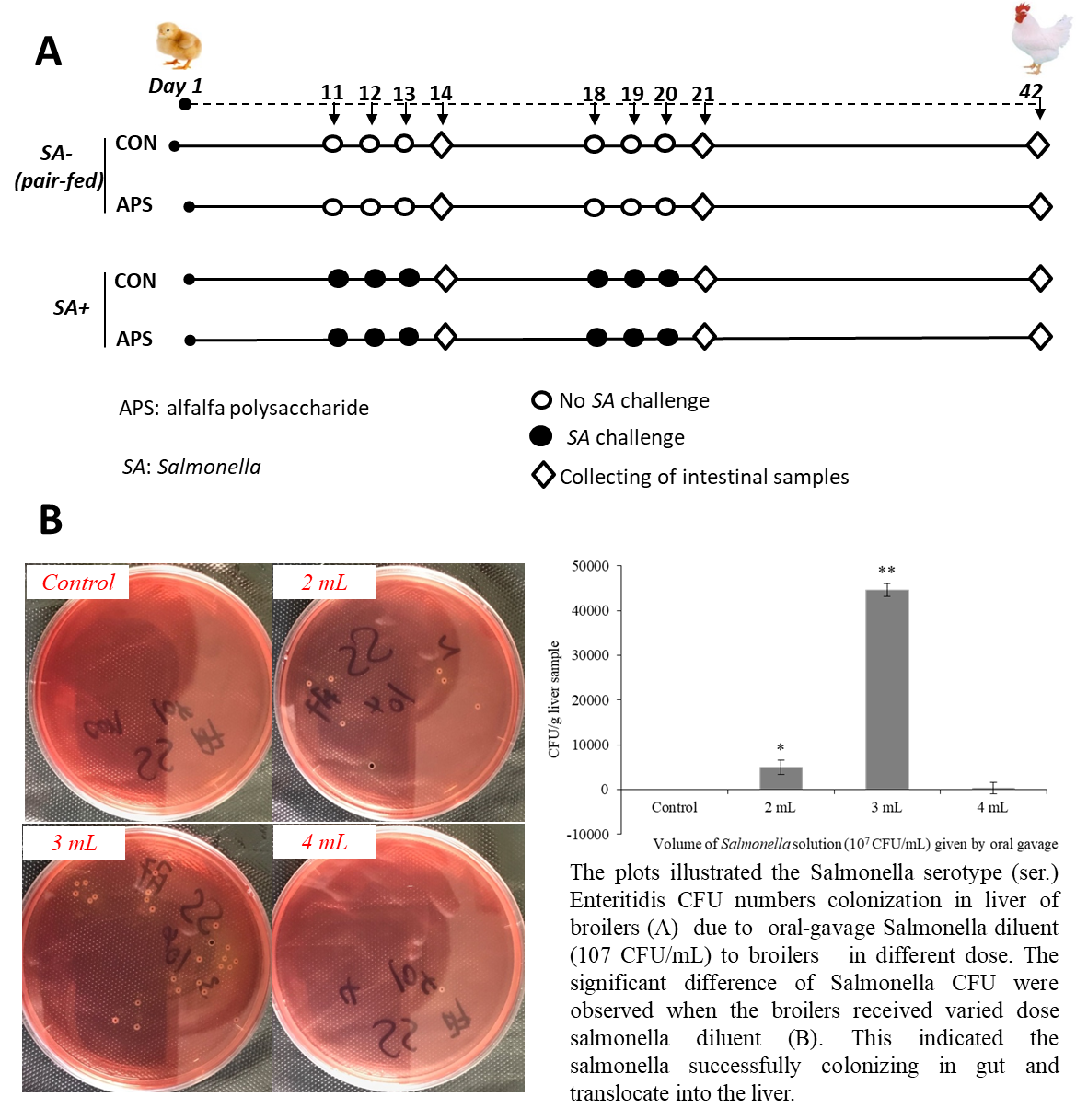


**Figure S1.** Profiles of *S. ser. Enteritidis* colonization and translocation in the livers of broilers challenged with different doses of *S. ser. Enteritidis* solution. APS, alfalfa polysaccharide; CON, control; SA, *Salmonella serotype (ser.) Enteritidis.*

**Supplement 2**

*Determination of intestinal tight junction protein mRNA expression*

Jejunum samples collected from broilers on day 42 and homogenized using normal saline. Total RNA was isolated from each jejunum sample using TRIzol reagent (Thermo Fisher, Waltham, Massachusetts, USA) according to the manufacturer’s instructions. The RNA concentration and purity were measured from OD 260/280 readings (ratio between 1.8 and 2.1) using a DS-11 ultra-micro spectrophotometer (DeNovix Inc., Wilmington, Delaware, USA). The cDNA was then generated by using a PrimeScript RT Master Mix Reagent Kit (TaKaRa Biotechnology, Beijing, China) according to the manufacturer’s instructions. Real-time PCR was conducted using an ABI 7500 Fast Real-time PCR system (Applied Biosystems, Grand Island, New York, USA) and a SYBR Green Premix Ex Taq kit (TaKaRa Biotechnology, Beijing, China) according to the manufacturers’ guidelines. The primer sequences used for the target and reference genes are shown in Table S1. The PCR mixture contained 10 μL of SYBR Premix Ex Taq, 0.4 μL of ROX Reference Dye II, 0.8 μL of forward primer, 0.8 μL of reverse primer, 2 μL of cDNA, and 6 μL of RNase-free water. Each sample was tested twice. The PCR protocol consisted of a pre-run cycle at 95°C for 30 s and 40 cycles of denaturation at 95°C for 5 s and annealing at 60°C for 34 s. The conditions for melting curve analysis were as follows: one cycle of denaturation at 95 °C for 15 s and annealing at 60 °C for 60 s followed by an increase in temperature from 60 °C to 95 °C at a rate of 0.5 °C /s (Luan et al., 2019). The relative levels of mRNA expression of the target genes were calculated using the 2^−ΔΔCT^ method, and β-actin was used as the reference gene (Cheng et al., 2019).

**Table S1.** List of qRT-PCR primers (Dong et al., 2016)

| Genes | Primer sequence | GenBank accession number |
| --- | --- | --- |
| MUC2 | Forward: 5’-CACCAACGGCAACTGAAATAGT-3’  Reverse: 5’-GCCAAACCATGGGTAACTCACA-3’ | XM 421035.2 |
| Claudin-1 | Forward: 5’-GCAGATCCAGTGCAAGGTGTA-3’  Reverse: 5’-CACTTCATGCCCGTCACAG-3’ | NM 001013611.2 |
| Occludin | Forward: 5’-CCGTAACCCCGAGTTGGAT-3’  Reverse: 5’-ATTGAGGCGGTCGTTGATG-3’ | NM 205128.1 |
| β-Actin | Forward: 5’-ATCCGGACCCTCCATTGTC-3’  Reverse: 5’-AGCCATGCCAATCTCGTCTT-3’ | NM 205518.2 |

**Supplement 3**

**Table S2.** The relative abundance of bacterial phyla, families, and genera in the cecal microbiota of broilers

| Phyla | Treatments | | | | SEM | *P-value* |
| --- | --- | --- | --- | --- | --- | --- |
|  | CON+SA | APS+SA | CON | APS |  |  |
| **Day 14** |  |  |  |  |  |  |
| *Firmicutes* | 0.8521 | 0.7795 | 0.8666 | 0.7210 | 0.0462 | 0.7202 |
| *Bacteroidetes* | 0.0504 | 0.0900 | 0.0645 | 0.0427 | 0.0211 | 0.9014 |
| *Tenericutes* | 0.0939 | 0.1228 | 0.0673 | 0.2350 | 0.0395 | 0.5141 |
| *Proteobacteria* | 0.0018 | 0.0039 | 0.0005 | 0.0007 | 0.0006 | 0.2106 |
| *Synergistetes* | 0.0001 | 0.0001 | 0.0002 | 0.0001 | 0.0000 | 0.3630 |
| *Fusobacteria* | 0.0000 | 0.0003 | 0.0001 | 0.0001 | 0.0001 | 0.4872 |
| **Day 21** |  |  |  |  |  |  |
| *Firmicutes* | 0.7381 | 0.7657 | 0.3669 | 0.2535 | 0.0798 | 0.0131 |
| *Bacteroidetes* | 0.1186 | 0.1092 | 0.6157 | 0.7126 | 0.0909 | 0.0013 |
| *Tenericutes* | 0.0223 | 0.0086 | 0.0083 | 0.0190 | 0.0052 | 0.7611 |
| *Proteobacteria* | 0.1121 | 0.1096 | 0.0062 | 0.0127 | 0.0336 | 0.5784 |
| *Synergistetes* | 0.0012 | 0.0004 | 0.0002 | 0.0004 | 0.0002 | 0.3552 |
| *Fusobacteria* | 0.0009 | 0.0000 | 0.0001 | 0.0001 | 0.0002 | 0.3634 |
| **Day 42** |  |  |  |  |  |  |
| *Firmicutes* | 0.3340 | 0.2003 | 0.2700 | 0.2399 | 0.0216 | 0.1508 |
| *Bacteroidetes* | 0.6028 | 0.7825 | 0.6793 | 0.7005 | 0.0245 | 0.0434 |
| *Tenericutes* | 0.0295 | 0.0014 | 0.0018 | 0.0013 | 0.0070 | 0.4456 |
| *Proteobacteria* | 0.0086 | 0.0078 | 0.0155 | 0.0117 | 0.0018 | 0.4855 |
| *Synergistetes* | 0.0096 | 0.0037 | 0.0179 | 0.0210 | 0.0047 | 0.6234 |
| *Fusobacteria* | 0.0086 | 0.0003 | 0.0006 | 0.0228 | 0.0043 | 0.2258 |

| Family | Treatments | | | | | | | | SEM | | *P-value* | |
| --- | --- | --- | --- | --- | --- | --- | --- | --- | --- | --- | --- | --- |
|  | CON+SA | | APS+SA | | CON | | APS | |  |  |  |  |
| **Day 14** |  | |  | |  | |  | |  | |  | |
| *Bacteroidaceae* | 0.0495 | | 0.0883 | | 0.0632 | | 0.0418 | | 0.0211 | | 0.9056 | |
| *Ruminococcaceae* | 0.4800 | | 0.2102 | | 0.1095 | | 0.1424 | | 0.0610 | | 0.1033 | |
| *Veillonellaceae* | 0.0343 | | 0.1059 | | 0.3933 | | 0.2123 | | 0.0651 | | 0.2400 | |
| *Lachnospiraceae* | 0.1245 | | 0.3518 | | 0.1772 | | 0.0801 | | 0.0446 | | 0.1308 | |
| *Lactobacillaceae* | 0.1742 | | 0.0648 | | 0.1455 | | 0.2448 | | 0.0358 | | 0.3999 | |
| *Porphyromonadaceae* | 0.0003 | | 0.0006 | | 0.0004 | | 0.0003 | | 0.0000 | | 0.3999 | |
| *Erysipelotrichaceae* | 0.0174 | | 0.0262 | | 0.0226 | | 0.0103 | | 0.0032 | | 0.3604 | |
| *Burkholderiaceae* | 0.0003 | | 0.0002 | | 0.0001 | | 0.0000 | | 0.0001 | | 0.0730 | |
| *Rikenellaceae* | 0.0004 | | 0.0007 | | 0.0007 | | 0.0004 | | 0.0001 | | 0.3316 | |
| *Prevotellaceae* | 0.0001 | | 0.0001 | | 0.0002 | | 0.0001 | | 0.0000 | | 0.8272 | |
| *Xanthomonadaceae* | 0.0001 | | 0.0002 | | 0.0000 | | 0.0000 | | 0.0000 | | 0.0402 | |
| *Bacteroidales_S24-7_group* | 0.0001 | | 0.0001 | | 0.0000 | | 0.0000 | | 0.0000 | | 0.0061 | |
| *Synergistaceae* | 0.0000 | | 0.0001 | | 0.0002 | | 0.0001 | | 0.0000 | | 0.3630 | |
| *Clostridiales_vadinBB60_group* | 0.0088 | | 0.0024 | | 0.0027 | | 0.0227 | | 0.0042 | | 0.2970 | |
| *Fusobacteriaceae* | 0.0000 | | 0.0003 | | 0.0001 | | 0.0001 | | 0.0001 | | 0.4872 | |
| *Sphingomonadaceae* | 0.0000 | | 0.0001 | | 0.0000 | | 0.0000 | | 0.0000 | | 0.0118 | |
| *Acidaminococcaceae* | 0.0080 | | 0.0111 | | 0.0122 | | 0.0051 | | 0.0029 | | 0.8671 | |
| *Others* | 0.0934 | | 0.1220 | | 0.0637 | | 0.2351 | | 0.0397 | | 0.5029 | |
| **Day 21** |  | |  | |  | |  | |  | |  | |
| *Bacteroidaceae* | 0.0368 | | 0.0507 | | 0.4129 | | 0.5997 | | 0.0765 | | 0.0002 | |
| *Ruminococcaceae* | 0.2518 | | 0.3372 | | 0.0430 | | 0.0986 | | 0.0486 | | 0.0934 | |
| *Veillonellaceae* | 0.0097 | | 0.0078 | | 0.0127 | | 0.0026 | | 0.0026 | | 0.6208 | |
| *Lachnospiraceae* | 0.2819 | | 0.1687 | | 0.0505 | | 0.0352 | | 0.0468 | | 0.2117 | |
| *Lactobacillaceae* | 0.1748 | | 0.1021 | | 0.2362 | | 0.1079 | | 0.0428 | | 0.7131 | |
| *Porphyromonadaceae* | 0.0140 | | 0.0085 | | 0.1078 | | 0.0491 | | 0.0183 | | 0.1975 | |
| *Erysipelotrichaceae* | 0.0096 | | 0.1323 | | 0.0124 | | 0.0042 | | 0.0202 | | 0.0349 | |
| *Burkholderiaceae* | 0.0571 | | 0.0615 | | 0.0001 | | 0.0014 | | 0.0189 | | 0.5549 | |
| *Rikenellaceae* | 0.0295 | | 0.0483 | | 0.0933 | | 0.0620 | | 0.0142 | | 0.5014 | |
| *Prevotellaceae* | 0.0375 | | 0.0010 | | 0.0013 | | 0.0008 | | 0.0068 | | 0.1207 | |
| *Xanthomonadaceae* | 0.0355 | | 0.0186 | | 0.0001 | | 0.0011 | | 0.0095 | | 0.5632 | |
| *Bacteroidales_S24-7_group* | 0.0005 | | 0.0004 | | 0.0001 | | 0.0000 | | 0.0001 | | 0.5747 | |
| *Synergistaceae* | 0.0012 | | 0.0004 | | 0.0002 | | 0.0004 | | 0.0002 | | 0.3552 | |
| *Clostridiales_vadinBB60_group* | 0.0003 | | 0.0002 | | 0.0008 | | 0.0008 | | 0.0002 | | 0.5702 | |
| *Fusobacteriaceae* | 0.0009 | | 0.0000 | | 0.0001 | | 0.0001 | | 0.0002 | | 0.3634 | |
| *Sphingomonadaceae* | 0.0041 | | 0.0155 | | 0.0001 | | 0.0012 | | 0.0038 | | 0.5188 | |
| *Acidaminococcaceae* | 0.0018 | | 0.0010 | | 0.0070 | | 0.0016 | | 0.0011 | | 0.1494 | |
| *Others* | 0.0254 | | 0.0132 | | 0.0098 | | 0.0206 | | 0.0053 | | 0.7808 | |
| **Day 42** |  | |  | |  | |  | |  | |  | |
| *Bacteroidaceae* | 0.4979 | | 0.6762 | | 0.5773 | | 0.5155 | | 0.0333 | | 0.2309 | |
| *Ruminococcaceae* | 0.0709 | | 0.0359 | | 0.0337 | | 0.0414 | | 0.0062 | | 0.0934 | |
| *Veillonellaceae* | 0.1518 | | 0.1100 | | 0.0773 | | 0.1048 | | 0.0155 | | 0.4465 | |
| *Lachnospiraceae* | 0.0579 | | 0.0173 | | 0.1107 | | 0.0654 | | 0.0129 | | 0.0508 | |
| *Lactobacillaceae* | 0.0078 | | 0.0063 | | 0.0075 | | 0.0047 | | 0.0007 | | 0.3647 | |
| *Porphyromonadaceae* | 0.0476 | | 0.0418 | | 0.0330 | | 0.0524 | | 0.0061 | | 0.7635 | |
| *Erysipelotrichaceae* | 0.0224 | | 0.0105 | | 0.0186 | | 0.0142 | | 0.0036 | | 0.7248 | |
| *Burkholderiaceae* | 0.0002 | | 0.0002 | | 0.0001 | | 0.0000 | | 0.0000 | | 0.0061 | |
| *Rikenellaceae* | 0.0269 | | 0.0104 | | 0.0114 | | 0.0486 | | 0.0057 | | 0.0241 | |
| *Prevotellaceae* | 0.0151 | | 0.0130 | | 0.0102 | | 0.0623 | | 0.0117 | | 0.3706 | |
| *Xanthomonadaceae* | 0.0002 | | 0.0001 | | 0.0001 | | 0.0000 | | 0.0000 | | 0.1456 | |
| *Bacteroidales_S24-7_group* | 0.0110 | | 0.0347 | | 0.0139 | | 0.0136 | | 0.0067 | | 0.6303 | |
| *Synergistaceae* | 0.0096 | | 0.0037 | | 0.0179 | | 0.0210 | | 0.0047 | | 0.6234 | |
| *Clostridiales_vadinBB60_group* | 0.0005 | | 0.0022 | | 0.0038 | | 0.0011 | | 0.0008 | | 0.6141 | |
| *Fusobacteriaceae* | 0.0086 | | 0.0003 | | 0.0006 | | 0.0228 | | 0.0043 | | 0.2258 | |
| *Sphingomonadaceae* | 0.0000 | | 0.0001 | | 0.0001 | | 0.0000 | | 0.0000 | | 0.3300 | |
| *Acidaminococcaceae* | 0.0147 | | 0.0161 | | 0.0158 | | 0.0071 | | 0.0026 | | 0.6414 | |
| *Others* | 0.0333 | | 0.0083 | | 0.0347 | | 0.0098 | | 0.0090 | | 0.6555 | |
| Genus | | Treatments | | | | | | | | SEM | | *P-value* |
|  |  | CON+SA | | APS+SA | | CON | | APS | |  |  |  |
| **Day 14** | |  | |  | |  | |  | |  | |  |
| *Bacteroides* | | 0.0495 | | 0.0883 | | 0.0632 | | 0.0418 | | 0.0211 | | 0.9056 |
| *Faecalibacterium* | | 0.2048 | | 0.0925 | | 0.0068 | | 0.0406 | | 0.0526 | | 0.6303 |
| *Megamonas* | | 0.0342 | | 0.1058 | | 0.3932 | | 0.2109 | | 0.0649 | | 0.2370 |
| *Subdoligranulum* | | 0.2132 | | 0.0272 | | 0.0087 | | 0.0585 | | 0.0490 | | 0.4940 |
| *Lactobacillus* | | 0.1742 | | 0.0648 | | 0.1455 | | 0.2448 | | 0.0358 | | 0.3999 |
| *Anaerotruncus* | | 0.0078 | | 0.0083 | | 0.0092 | | 0.0026 | | 0.0013 | | 0.2869 |
| *Barnesiella* | | 0.0001 | | 0.0003 | | 0.0001 | | 0.0001 | | 0.0000 | | 0.0969 |
| *Erysipelatoclostridium* | | 0.0164 | | 0.0237 | | 0.0183 | | 0.0072 | | 0.0033 | | 0.3915 |
| *[Ruminococcus]_torques_group* | | 0.0158 | | 0.1119 | | 0.0278 | | 0.0137 | | 0.0180 | | 0.1553 |
| *Ralstonia* | | 0.0002 | | 0.0002 | | 0.0001 | | 0.0000 | | 0.0000 | | 0.1598 |
| *Eisenbergiella* | | 0.0021 | | 0.0165 | | 0.0106 | | 0.0071 | | 0.0031 | | 0.4591 |
| *Alistipes* | | 0.0004 | | 0.0007 | | 0.0006 | | 0.0004 | | 0.0001 | | 0.3374 |
| *Sellimonas* | | 0.0100 | | 0.0167 | | 0.0093 | | 0.0047 | | 0.0021 | | 0.2890 |
| *Ruminococcaceae_UCG-014* | | 0.0074 | | 0.0230 | | 0.0098 | | 0.0128 | | 0.0037 | | 0.5132 |
| *Parabacteroides* | | 0.0001 | | 0.0001 | | 0.0002 | | 0.0002 | | 0.0000 | | 0.7520 |
| *Stenotrophomonas* | | 0.0001 | | 0.0002 | | 0.0000 | | 0.0000 | | 0.0000 | | 0.1388 |
| *Prevotellaceae_UCG-001* | | 0.0000 | | 0.0000 | | 0.0001 | | 0.0001 | | 0.0000 | | 0.0553 |
| *Lachnospiraceae_NK4A136_group* | | 0.0124 | | 0.0364 | | 0.0490 | | 0.0072 | | 0.0092 | | 0.3614 |
| *Synergistes* | | 0.0000 | | 0.0001 | | 0.0002 | | 0.0001 | | 0.0000 | | 0.3630 |
| *Butyricicoccus* | | 0.0080 | | 0.0350 | | 0.0102 | | 0.0056 | | 0.0049 | | 0.1006 |
| *Fusobacterium* | | 0.0000 | | 0.0001 | | 0.0001 | | 0.0001 | | 0.0000 | | 0.4872 |
| *Sphingomonas* | | 0.0000 | | 0.0001 | | 0.0000 | | 0.0000 | | 0.0000 | | 0.0118 |
| *Ruminococcaceae_NK4A214_group* | | 0.0003 | | 0.0000 | | 0.0001 | | 0.0001 | | 0.0001 | | 0.3700 |
| *Phascolarctobacterium* | | 0.0080 | | 0.0111 | | 0.0122 | | 0.0051 | | 0.0029 | | 0.8671 |
| *unidentified_Ruminococcaceae* | | 0.0058 | | 0.0032 | | 0.0112 | | 0.0015 | | 0.0025 | | 0.5923 |
| *Butyricimonas* | | 0.0000 | | 0.0000 | | 0.0001 | | 0.0000 | | 0.0000 | | 0.8592 |
| *[Eubacterium]_coprostanoligenes_group* | | 0.0009 | | 0.0014 | | 0.0028 | | 0.0008 | | 0.0005 | | 0.4995 |
| *Ruminiclostridium_5* | | 0.0046 | | 0.0041 | | 0.0100 | | 0.0022 | | 0.0017 | | 0.4769 |
| *Prevotellaceae_NK3B31_group* | | 0.0001 | | 0.0000 | | 0.0000 | | 0.0000 | | 0.0000 | | 0.5957 |
| *Others* | | 0.1855 | | 0.2695 | | 0.1581 | | 0.2977 | | 0.0439 | | 0.6959 |
| **Day 21** | |  | |  | |  | |  | |  | |  |
| *Bacteroides* | | 0.0368 | | 0.0507 | | 0.4129 | | 0.5997 | | 0.0765 | | 0.0002 |
| *Faecalibacterium* | | 0.1914 | | 0.1584 | | 0.0190 | | 0.0139 | | 0.0428 | | 0.3560 |
| *Megamonas* | | 0.0088 | | 0.0069 | | 0.0096 | | 0.0021 | | 0.0024 | | 0.7626 |
| *Subdoligranulum* | | 0.0097 | | 0.0062 | | 0.0022 | | 0.0076 | | 0.0011 | | 0.0423 |
| *Lactobacillus* | | 0.1748 | | 0.1021 | | 0.2362 | | 0.1079 | | 0.0428 | | 0.7131 |
| *Anaerotruncus* | | 0.0068 | | 0.0903 | | 0.0011 | | 0.0010 | | 0.0212 | | 0.4067 |
| *Barnesiella* | | 0.0098 | | 0.0050 | | 0.0419 | | 0.0273 | | 0.0076 | | 0.3175 |
| *Erysipelatoclostridium* | | 0.0086 | | 0.1310 | | 0.0109 | | 0.0033 | | 0.0202 | | 0.0354 |
| *[Ruminococcus]_torques_group* | | 0.0344 | | 0.0144 | | 0.0103 | | 0.0085 | | 0.0067 | | 0.5542 |
| *Ralstonia* | | 0.0571 | | 0.0615 | | 0.0001 | | 0.0014 | | 0.0189 | | 0.5553 |
| *Eisenbergiella* | | 0.0599 | | 0.0038 | | 0.0011 | | 0.0017 | | 0.0146 | | 0.4506 |
| *Alistipes* | | 0.0293 | | 0.0483 | | 0.0922 | | 0.0605 | | 0.0142 | | 0.5192 |
| *Sellimonas* | | 0.0057 | | 0.0489 | | 0.0038 | | 0.0048 | | 0.0111 | | 0.4466 |
| *Ruminococcaceae_UCG-014* | | 0.0126 | | 0.0239 | | 0.0030 | | 0.0498 | | 0.0116 | | 0.5849 |
| *Parabacteroides* | | 0.0015 | | 0.0024 | | 0.0581 | | 0.0036 | | 0.0103 | | 0.1227 |
| *Stenotrophomonas* | | 0.0355 | | 0.0186 | | 0.0001 | | 0.0011 | | 0.0094 | | 0.5632 |
| *Prevotellaceae_UCG-001* | | 0.0245 | | 0.0002 | | 0.0013 | | 0.0006 | | 0.0061 | | 0.4630 |
| *Lachnospiraceae_NK4A136_group* | | 0.0003 | | 0.0001 | | 0.0005 | | 0.0004 | | 0.0001 | | 0.0353 |
| *Synergistes* | | 0.0012 | | 0.0004 | | 0.0002 | | 0.0004 | | 0.0002 | | 0.3552 |
| *Butyricicoccus* | | 0.0014 | | 0.0038 | | 0.0041 | | 0.0021 | | 0.0006 | | 0.3107 |
| *Fusobacterium* | | 0.0008 | | 0.0000 | | 0.0001 | | 0.0001 | | 0.0002 | | 0.5133 |
| *Sphingomonas* | | 0.0041 | | 0.0155 | | 0.0001 | | 0.0012 | | 0.0038 | | 0.5188 |
| *Ruminococcaceae_NK4A214_group* | | 0.0006 | | 0.0005 | | 0.0003 | | 0.0136 | | 0.0033 | | 0.4572 |
| *Phascolarctobacterium* | | 0.0018 | | 0.0010 | | 0.0070 | | 0.0016 | | 0.0011 | | 0.1494 |
| *unidentified_Ruminococcaceae* | | 0.0011 | | 0.0009 | | 0.0007 | | 0.0006 | | 0.0002 | | 0.8538 |
| *Butyricimonas* | | 0.0007 | | 0.0001 | | 0.0041 | | 0.0069 | | 0.0013 | | 0.1937 |
| *[Eubacterium]_coprostanoligenes_group* | | 0.0031 | | 0.0145 | | 0.0015 | | 0.0007 | | 0.0022 | | 0.0742 |
| *Ruminiclostridium_5* | | 0.0028 | | 0.0052 | | 0.0012 | | 0.0007 | | 0.0011 | | 0.5593 |
| *Prevotellaceae_NK3B31_group* | | 0.0129 | | 0.0000 | | 0.0000 | | 0.0000 | | 0.0018 | | 0.0007 |
| *Others* | | 0.2054 | | 0.1301 | | 0.0440 | | 0.0509 | | 0.0402 | | 0.5006 |
| **Day 42** | |  | |  | |  | |  | |  | |  |
| *Bacteroides* | | 0.4979 | | 0.6762 | | 0.5773 | | 0.5155 | | 0.0333 | | 0.2309 |
| *Faecalibacterium* | | 0.0321 | | 0.0233 | | 0.0057 | | 0.0151 | | 0.0046 | | 0.2168 |
| *Megamonas* | | 0.1485 | | 0.1074 | | 0.0743 | | 0.1031 | | 0.0154 | | 0.4479 |
| *Subdoligranulum* | | 0.0012 | | 0.0011 | | 0.0033 | | 0.0007 | | 0.0005 | | 0.2942 |
| *Lactobacillus* | | 0.0078 | | 0.0063 | | 0.0075 | | 0.0047 | | 0.0007 | | 0.3647 |
| *Anaerotruncus* | | 0.0015 | | 0.0010 | | 0.0012 | | 0.0006 | | 0.0002 | | 0.3563 |
| *Barnesiella* | | 0.0083 | | 0.0040 | | 0.0044 | | 0.0045 | | 0.0010 | | 0.4665 |
| *Erysipelatoclostridium* | | 0.0196 | | 0.0089 | | 0.0127 | | 0.0041 | | 0.0032 | | 0.4356 |
| *[Ruminococcus]_torques_group* | | 0.0068 | | 0.0026 | | 0.0384 | | 0.0286 | | 0.0075 | | 0.2858 |
| *Ralstonia* | | 0.0002 | | 0.0002 | | 0.0001 | | 0.0000 | | 0.0000 | | 0.0086 |
| *Eisenbergiella* | | 0.0032 | | 0.0003 | | 0.0005 | | 0.0003 | | 0.0007 | | 0.4661 |
| *Alistipes* | | 0.0265 | | 0.0104 | | 0.0109 | | 0.0485 | | 0.0057 | | 0.0235 |
| *Sellimonas* | | 0.0022 | | 0.0024 | | 0.0017 | | 0.0026 | | 0.0005 | | 0.9402 |
| *Ruminococcaceae_UCG-014* | | 0.0169 | | 0.0011 | | 0.0021 | | 0.0009 | | 0.0039 | | 0.4564 |
| *Parabacteroides* | | 0.0109 | | 0.0237 | | 0.0190 | | 0.0256 | | 0.0062 | | 0.8780 |
| *Stenotrophomonas* | | 0.0002 | | 0.0001 | | 0.0000 | | 0.0000 | | 0.0000 | | 0.4635 |
| *Prevotellaceae_UCG-001* | | 0.0073 | | 0.0096 | | 0.0064 | | 0.0368 | | 0.0076 | | 0.4804 |
| *Lachnospiraceae_NK4A136_group* | | 0.0037 | | 0.0002 | | 0.0004 | | 0.0003 | | 0.0008 | | 0.3875 |
| *Synergistes* | | 0.0096 | | 0.0037 | | 0.0179 | | 0.0210 | | 0.0047 | | 0.6234 |
| *Butyricicoccus* | | 0.0006 | | 0.0004 | | 0.0021 | | 0.0008 | | 0.0004 | | 0.3820 |
| *Fusobacterium* | | 0.0086 | | 0.0000 | | 0.0003 | | 0.0228 | | 0.0044 | | 0.2177 |
| *Sphingomonas* | | 0.0000 | | 0.0000 | | 0.0000 | | 0.0000 | | 0.0000 | | 0.8018 |
| *Ruminococcaceae_NK4A214_group* | | 0.0038 | | 0.0002 | | 0.0003 | | 0.0002 | | 0.0007 | | 0.2557 |
| *Phascolarctobacterium* | | 0.0147 | | 0.0161 | | 0.0158 | | 0.0071 | | 0.0026 | | 0.6414 |
| *unidentified_Ruminococcaceae* | | 0.0002 | | 0.0005 | | 0.0005 | | 0.0002 | | 0.0001 | | 0.5815 |
| *Butyricimonas* | | 0.0124 | | 0.0044 | | 0.0022 | | 0.0092 | | 0.0022 | | 0.3811 |
| *[Eubacterium]_coprostanoligenes_group* | | 0.0009 | | 0.0006 | | 0.0003 | | 0.0012 | | 0.0002 | | 0.6792 |
| *Ruminiclostridium_5* | | 0.0016 | | 0.0003 | | 0.0003 | | 0.0002 | | 0.0002 | | 0.0205 |
| *Prevotellaceae_NK3B31_group* | | 0.0023 | | 0.0009 | | 0.0002 | | 0.0014 | | 0.0004 | | 0.1807 |
| *Others* | | 0.0956 | | 0.0608 | | 0.1253 | | 0.0889 | | 0.0147 | | 0.5483 |

**Table S3.** The relative abundance of discrepant bacteria among groups.

| **Items** | **Treatments** | | | | | | **Age** |
| --- | --- | --- | --- | --- | --- | --- | --- |
| **Phylum** |  |  |  |  |  |  |  |
| CON *vs.* CON+SA | CON | CON | CON | CON+SA | CON+SA | CON+SA |  |
| *Proteobacteria* | 0.0005 | 0.0006 | 0.000294 | 0.00171 | 0.001175 | 0.00203 | d14 |
| *Bacteroidetes* | 0.7151 | 0.740723 | 0.39104 | 0.117496 | 0.065614 | 0.172584 | d21 |
| CON+SA *vs.* APS+SA | CON+SA | CON+SA | CON+SA | APS+SA | APS+SA | APS+SA |  |
| *Bacteroidetes* | 0.537229 | 0.647805 | 0.619967 | 0.743047 | 0.843552 | 0.765702 | d42 |
| APS *vs.* APS+SA | APS | APS | APS | APS+SA | APS+SA | APS+SA |  |
| *Firmicutes* | 0.260132 | 0.084182 | 0.41861 | 0.756699 | 0.656862 | 0.885977 | d21 |
| *Bacteroidetes* | 0.734498 | 0.876519 | 0.523844 | 0.226657 | 0.011995 | 0.086639 | d21 |
| *Actinobacteria* | 0.000534 | 0.000748 | 0.000134 | 0.002618 | 0.002324 | 0.001443 | d42 |
| **Family** |  |  |  |  |  |  |  |
| CON *vs.* CON+SA | CON | CON | CON | CON+SA | CON+SA | CON+SA |  |
| *Bacteroidaceae* | 0.488312 | 0.429457 | 0.319655 | 0.043093 | 0.031471 | 0.035772 | d21 |
| *Marinifilaceae* | 0.004675 | 0.006919 | 0.004889 | 0.000561 | 0.000801 | 0.001363 | d21 |
| *Erysipelotrichaceae* | 0.02351 | 0.010473 | 0.021773 | 0.013064 | 0.00569 | 0.04881 | d42 |
| CON+SA *vs.* APS+SA | CON+SA | CON+SA | CON+SA | APS+SA | APS+SA | APS+SA |  |
| *Bacteroidaceae* | 0.413508 | 0.542732 | 0.537175 | 0.62312 | 0.757741 | 0.65916 | d42 |
| *Ruminococcaceae* | 0.07045 | 0.062702 | 0.078598 | 0.044348 | 0.032273 | 0.02912 | d42 |
| *Lachnospiraceae* | 0.060966 | 0.050653 | 0.064545 | 0.019316 | 0.010633 | 0.021239 | d42 |
| *Burkholderiaceae* | 0.007293 | 0.007935 | 0.004462 | 0.001443 | 0.000882 | 0.001469 | d42 |
| *Barnesiellaceae* | 0.012396 | 0.012476 | 0.008709 | 0.004408 | 0.003313 | 0.007801 | d42 |
| APS *vs.* APS+SA | APS+SA | APS+SA | APS+SA | APS | APS | APS |  |
| *Bacteroidaceae* | 0.090112 | 0.002351 | 0.059576 | 0.640993 | 0.747642 | 0.412412 | d21 |
| *Rikenellaceae* | 0.004114 | 0.002778 | 0.024872 | 0.031044 | 0.046432 | 0.067297 | d42 |
| **Genus** |  |  |  |  |  |  |  |
| APS *vs.* APS+SA | APS1 | APS1 | APS1 | APS+SA1 | APS+SA1 | APS+SA1 |  |
| *unidentified_Lachnospiraceae (d 14)* | 0.008335 | 0.041169 | 0.018674 | 0.079186 | 0.079693 | 0.050065 | 14 |
| *Sellimonas (d 14)* | 0.001496 | 0.002057 | 0.010526 | 0.012877 | 0.018781 | 0.018701 | 14 |
| *Bacteroides (d 21)* | 0.640993 | 0.747642 | 0.412412 | 0.090112 | 0.002351 | 0.059576 | 21 |
| *Barnesiella (d 21)* | 0.026449 | 0.017419 | 0.037295 | 0.008709 | 0.000695 | 0.005824 |  |
| *Alistipes (d 42)* | 0.03091 | 0.046325 | 0.067083 | 0.004088 | 0.002778 | 0.024819 | 42 |
| CON *vs.* CON+SA | CON | CON | CON | CON+SA | CON+SA | CON+SA |  |
| *Megamonas (d 14)* | 0.504421 | 0.235046 | 0.443135 | 0.001202 | 0.095082 | 0.002378 | 14 |
| *Bacteroides (d 21)* | 0.488312 | 0.429457 | 0.319655 | 0.043093 | 0.031471 | 0.035772 | 21 |
| *unidentified_Enterobacteriaceae (d 42)* | 0.000882 | 0.00155 | 0.001363 | 0.000214 | 5.34E-05 | 2.67E-05 | 42 |
| CON+SA *vs.* APS+SA | CON+SA | CON+SA | CON+SA | APS+SA | APS+SA | APS+SA |  |
| *unidentified_Lachnospiraceae* | 0.018514 | 0.00545 | 0.031177 | 0.079186 | 0.079693 | 0.050065 | 14 |
| *Bacteroides* | 0.413508 | 0.542732 | 0.537175 | 0.62312 | 0.757741 | 0.65916 | 42 |
| *Sutterella* | 0.002137 | 0.002378 | 0.003233 | 0.000187 | 0 | 0.000721 |  |
| CON *vs.* APS | CON | CON | CON | APS | APS | APS |  |
| *unidentified_Enterobacteriaceae* | 0.000882 | 0.00155 | 0.001363 | 0.000481 | 2.67E-05 | 0.00016 | 42 |

**Table S4.** The relative abundance of functional genes of cecal microbiota of broilers in different ages post *S. ser. Enteritidis*-infection

| **Metabolic Pathways (Day 14)** | **CON** | **APS** | **CON+SA** | **APS+SA** | **SEM** | ***P*-values** |
| --- | --- | --- | --- | --- | --- | --- |
| Carbohydrate_metabolism | 0.0994^b^ | 0.1067^ab^ | 0.1101^a^ | 0.1072^ab^ | 0.0013 | 0.1007 |
| Replication_and_repair | 0.1028^a^ | 0.1005^b^ | 0.0998b | 0.1002^b^ | 0.0003 | 0.0444 |
| Translation | 0.1013^a^ | 0.0982^ab^ | 0.0971b | 0.0972^b^ | 0.0006 | 0.1128 |
| Membrane_transport | 0.1011^b^ | 0.1036^ab^ | 0.1047a | 0.1042^ab^ | 0.0005 | 0.1388 |
| Amino_acid_metabolism | 0.0885^a^ | 0.0870^ab^ | 0.0861b | 0.0869^ab^ | 0.0003 | 0.0714 |
| Energy_metabolism | 0.0455^a^ | 0.0424^ab^ | 0.0414b | 0.0435^ab^ | 0.0006 | 0.1538 |
| Nucleotide_metabolism | 0.0436^a^ | 0.0429^ab^ | 0.0425b | 0.0430^ab^ | 0.0001 | 0.0511 |
| Glycan_biosynthesis_and_metabolism | 0.0340^a^ | 0.0313^ab^ | 0.0292b | 0.0297^b^ | 0.0006 | 0.0961 |
| Metabolism_of_cofactors_and_vitamins | 0.0378^a^ | 0.0337^ab^ | 0.0317b | 0.0332^ab^ | 0.0008 | 0.1287 |
| Transport_and_catabolism | 0.0243 | 0.0256 | 0.0264 | 0.0250 | 0.0003 | 0.1798 |
| Signal_transduction | 0.0307 | 0.0319 | 0.0320 | 0.0324 | 0.0003 | 0.3569 |
| Folding,_sorting_and_degradation | 0.0261^a^ | 0.0250^ab^ | 0.0246b | 0.0252^ab^ | 0.0002 | 0.1654 |
| Cell_motility | 0.0466 | 0.0396 | 0.0356 | 0.0369 | 0.0017 | 0.1909 |
| Lipid_metabolism | 0.0191^b^ | 0.0215^ab^ | 0.0230a | 0.0223^ab^ | 0.0005 | 0.0874 |
| Enzyme_families | 0.0191 | 0.0209 | 0.0217 | 0.0219 | 0.0005 | 0.2171 |
| Cellular_community___prokaryotes | 0.0195 | 0.0193 | 0.0192 | 0.0192 | 0.0001 | 0.1991 |
| Transcription | 0.0160^b^ | 0.0186^ab^ | 0.0198a | 0.0188^ab^ | 0.0005 | 0.0876 |
| Biosynthesis_of_other_secondary_metabolites | 0.0125^b^ | 0.0133^ab^ | 0.0139a | 0.0134^ab^ | 0.0001 | 0.0357 |
| Metabolism | 0.0115^b^ | 0.0146^ab^ | 0.0168a | 0.0156^a^ | 0.0006 | 0.0594 |
| Cellular_processes_and_signaling | 0.0119 | 0.0130 | 0.0134 | 0.0133 | 0.0002 | 0.1565 |
| Metabolism_of_other_amino_acids | 0.0123^b^ | 0.0131^ab^ | 0.0135a | 0.0133^ab^ | 0.0001 | 0.0907 |
| Genetic_information_processing | 0.0124 | 0.0125 | 0.0123 | 0.0127 | 0.0003 | 0.9798 |
| Drug_resistance | 0.0087 | 0.0087 | 0.0084 | 0.0087 | 0.0001 | 0.5333 |
| Poorly_characterized | 0.0101 | 0.0103 | 0.0101 | 0.0099 | 0.0001 | 0.5277 |
| Cell_growth_and_death | 0.0092 | 0.0090 | 0.0091 | 0.0092 | 0.0010 | 0.3097 |
| Metabolism_of_terpenoids_and_polyketides | 0.0086 | 0.0090 | 0.0094 | 0.0092 | 0.0001 | 0.2667 |
| Endocrine_system | 0.0078 | 0.0001 | 0.0078 | 0.0076 | 0.0010 | 0.3929 |
| Xenobiotics_biodegradation_and_metabolism | 0.0073^b^ | 0.0093^ab^ | 0.0103a | 0.0094^ab^ | 0.0004 | 0.1055 |
| Infectious_diseases | 0.0086^a^ | 0.0074^ab^ | 0.0065b | 0.0070^ab^ | 0.0003 | 0.0830 |
| Cancers | 0.0056^a^ | 0.0051^ab^ | 0.0048b | 0.0052^ab^ | 0.0001 | 0.0566 |

| **Metabolic pathways (Day 21)** | **CON** | **APS** | **CON+SA** | **APS+SA** | **SEM** | ***P*-values** |
| --- | --- | --- | --- | --- | --- | --- |
| Carbohydrate_metabolism | 0.1209^a^ | 0.1201^a^ | 0.1109^b^ | 0.1108^b^ | 0.0008 | 0.0018 |
| Replication_and_repair | 0.0922^b^ | 0.0926^b^ | 0.0993^a^ | 0.0984^a^ | 0.0005 | 0.0012 |
| Translation | 0.0893^b^ | 0.0894^b^ | 0.0959^a^ | 0.0951^a^ | 0.0005 | 0.0019 |
| Membrane_transport | 0.0835^b^ | 0.0825^b^ | 0.1014^a^ | 0.0996^a^ | 0.0010 | 0.0002 |
| Amino_acid_metabolism | 0.0857^b^ | 0.0856^b^ | 0.0867^ab^ | 0.0879^a^ | 0.0002 | 0.0096 |
| Energy_metabolism | 0.0459^a^ | 0.0460^a^ | 0.0435^b^ | 0.0428^b^ | 0.0002 | 0.0012 |
| Nucleotide_metabolism | 0.0417 | 0.0418 | 0.0430 | 0.0423 | 0.0002 | 0.1473 |
| Glycan_biosynthesis_and_metabolism | 0.0535^a^ | 0.0527^a^ | 0.0319^b^ | 0.0316^b^ | 0.0011 | <0.0001 |
| Metabolism_of_cofactors_and_vitamins | 0.0338^a^ | 0.0339^a^ | 0.0322^b^ | 0.0323^b^ | 0.0001 | 0.0042 |
| Transport_and_catabolism | 0.0331^a^ | 0.0332^a^ | 0.0268^b^ | 0.0272^b^ | 0.0004 | 0.0010 |
| Signal_transduction | 0.0249^b^ | 0.0252^b^ | 0.0305^a^ | 0.0305^a^ | 0.0007 | 0.0348 |
| Folding,_sorting_and_degradation | 0.0283^a^ | 0.0282^a^ | 0.0256^b^ | 0.0249^b^ | 0.0002 | 0.0009 |
| Cell_motility | 0.0095^b^ | 0.0100^b^ | 0.0291^a^ | 0.0288^a^ | 0.0012 | 0.0005 |
| Lipid_metabolism | 0.0289^a^ | 0.0288^a^ | 0.0241^b^ | 0.0247^b^ | 0.0003 | 0.0002 |
| Enzyme_families | 0.0268^a^ | 0.0271^a^ | 0.0232^b^ | 0.0228^b^ | 0.0002 | 0.0001 |
| Cellular_community___prokaryotes | 0.0165^b^ | 0.0165^b^ | 0.0186^a^ | 0.0187^a^ | 0.0002 | 0.0023 |
| Transcription | 0.0169^b^ | 0.0171^b^ | 0.0193^a^ | 0.0195^a^ | 0.0001 | 0.0001 |
| Biosynthesis_of_other_secondary_metabolites | 0.0180^a^ | 0.0182^a^ | 0.0142^b^ | 0.0143^b^ | 0.0002 | 0.0003 |
| Metabolism | 0.0160^b^ | 0.0161^b^ | 0.0167ab | 0.0175^a^ | 0.0002 | 0.0332 |
| Cellular_processes_and_signaling | 0.0176^a^ | 0.0176^a^ | 0.0141b | 0.0143^b^ | 0.0002 | <0.0001 |
| Metabolism_of_other_amino_acids | 0.0167^a^ | 0.0168^a^ | 0.0140^b^ | 0.0143^b^ | 0.0002 | 0.0002 |
| Genetic_information_processing | 0.0089^b^ | 0.0092^b^ | 0.0121^a^ | 0.0135^a^ | 0.0004 | 0.0042 |
| Drug_resistance | 0.0128^a^ | 0.0127^a^ | 0.0093^b^ | 0.0089^b^ | 0.0002 | <0.0001 |
| Poorly_characterized | 0.0112^a^ | 0.0111^a^ | 0.0099^b^ | 0.0099^b^ | 0.0001 | 0.0003 |
| Cell_growth_and_death | 0.0100^a^ | 0.0100^a^ | 0.0094^b^ | 0.0092^b^ | 0.0001 | 0.0019 |
| Metabolism_of_terpenoids_and_polyketides | 0.0100^ab^ | 0.1001^ab^ | 0.0097^b^ | 0.0104^a^ | 0.0001 | 0.0964 |
| Endocrine_system | 0.0087^a^ | 0.0088^a^ | 0.0078^b^ | 0.0079^b^ | 0.0001 | 0.0017 |
| Xenobiotics_biodegradation_and_metabolism | 0.0059^b^ | 0.0060^b^ | 0.0096^a^ | 0.0109^a^ | 0.0003 | 0.0003 |
| Infectious_diseases | 0.0064 | 0.0063 | 0.0064 | 0.0064 | 0.0001 | 0.8182 |
| Cancers | 0.0050 | 0.0049 | 0.0052 | 0.0053 | 0.0001 | 0.1968 |
| Immune_system | 0.0032 | 0.0033 | 0.0033 | 0.0033 | 0.0001 | 0.3305 |
| Aging | 0.0034 | 0.0034 | 0.0032 | 0.0033 | 0.0001 | 0.1553 |
| Endocrine_and_metabolic_diseases | 0.0028^b^ | 0.0028^b^ | 0.0033^a^ | 0.0034^a^ | 0.0001 | 0.0010 |
| Nervous_system | 0.0032^a^ | 0.0032^a^ | 0.0028^b^ | 0.0028^b^ | 0.0001 | 0.0010 |
| Environmental_adaptation | 0.0015^b^ | 0.0015^b^ | 0.0017^a^ | 0.0016^ab^ | 0.0001 | 0.0400 |
| Signaling_molecules_and_interaction | 0.0025^a^ | 0.0025^a^ | 0.0012^b^ | 0.0011^b^ | 0.0001 | 0.0002 |
| Cardiovascular_diseases | 0.0014^a^ | 0.0014^a^ | 0.0013^b^ | 0.0014^ab^ | 0.0001 | 0.0430 |
| Neurodegenerative_diseases | 0.0008^b^ | 0.0007^b^ | 0.0008^ab^ | 0.0009^a^ | 0.0001 | 0.0266 |
| Digestive_system | 0.0014^a^ | 0.0013^a^ | 0.0003^b^ | 0.0002^b^ | 0.0001 | <0.0001 |

| **Metabolic pathways (Day 42)** | **CON** | **APS** | **CON+SA** | **APS+SA** | **SEM** | ***P*-values** |
| --- | --- | --- | --- | --- | --- | --- |
| Carbohydrate_metabolism | 0.1172 | 0.1162 | 0.1140 | 0.1171 | 0.0006 | 0.2902 |
| Replication_and_repair | 0.0938 | 0.0952 | 0.0954 | 0.0942 | 0.0004 | 0.4583 |
| Translation | 0.0915 | 0.0924 | 0.0930 | 0.0915 | 0.0004 | 0.4440 |
| Membrane_transport | 0.0850 | 0.0842 | 0.0870 | 0.0839 | 0.0006 | 0.3251 |
| Amino_acid_metabolism | 0.0866 | 0.0861 | 0.0864 | 0.0858 | 0.0001 | 0.3115 |
| Energy_metabolism | 0.0465 | 0.0466 | 0.0464 | 0.0466 | 0.0001 | 0.6834 |
| Nucleotide_metabolism | 0.0423 | 0.0426 | 0.0424 | 0.0424 | 0.0001 | 0.6948 |
| Glycan_biosynthesis_and_metabolism | 0.0506 | 0.0507 | 0.0485 | 0.0520 | 0.0007 | 0.4234 |
| Metabolism_of_cofactors_and_vitamins | 0.0349 | 0.0351 | 0.0356 | 0.0355 | 0.0002 | 0.5892 |
| Transport_and_catabolism | 0.0318 | 0.0318 | 0.0308 | 0.0322 | 0.0003 | 0.3480 |
| Signal_transduction | 0.0257 | 0.0251 | 0.0261 | 0.0249 | 0.0002 | 0.2625 |
| Folding,_sorting_and_degradation | 0.0282 | 0.0282 | 0.0279 | 0.0284 | 0.0001 | 0.3847 |
| Cell_motility | 0.0144 | 0.0156 | 0.0200 | 0.0145 | 0.0011 | 0.2730 |
| Lipid_metabolism | 0.0273 | 0.0268 | 0.0260 | 0.0274 | 0.0003 | 0.3555 |
| Enzyme_families | 0.0257 | 0.0257 | 0.0247 | 0.0257 | 0.0003 | 0.4861 |
| Cellular_community___prokaryotes | 0.0170 | 0.0169 | 0.0173 | 0.0167 | 0.0001 | 0.1831 |
| Transcription | 0.0166 | 0.0165 | 0.0165 | 0.0164 | 0.0001 | 0.8402 |
| Biosynthesis_of_other_secondary_metabolites | 0.0173 | 0.0172 | 0.0167 | 0.0175 | 0.0002 | 0.5076 |
| Metabolism | 0.0151 | 0.0149 | 0.0145 | 0.0149 | 0.0001 | 0.6390 |
| Cellular_processes_and_signaling | 0.0167 | 0.0165 | 0.0160 | 0.0168 | 0.0002 | 0.3203 |
| Metabolism_of_other_amino_acids | 0.0162 | 0.0160 | 0.0157 | 0.0164 | 0.0001 | 0.3715 |
| Genetic_information_processing | 0.0092 | 0.0093 | 0.0096 | 0.0089 | 0.0002 | 0.4704 |
| Drug_resistance | 0.0123 | 0.0122 | 0.0117 | 0.0124 | 0.0001 | 0.2861 |
| Poorly_characterized | 0.0109 | 0.0109 | 0.0109 | 0.0112 | 0.0001 | 0.3382 |
| Cell_growth_and_death | 0.0099 | 0.0101 | 0.0099 | 0.0101 | 0.0001 | 0.3802 |
| Metabolism_of_terpenoids_and_polyketides | 0.0097 | 0.0097 | 0.0096 | 0.0097 | 0.0001 | 0.8163 |
| Endocrine_system | 0.0086 | 0.0087 | 0.0086 | 0.0088 | 0.0001 | 0.4342 |
| Xenobiotics_biodegradation_and_metabolism | 0.0060 | 0.0057 | 0.0060 | 0.0055 | 0.0001 | 0.3842 |
| Infectious_diseases | 0.0067 | 0.0068 | 0.0071 | 0.0068 | 0.0001 | 0.4706 |
| Cancers | 0.0051 | 0.0051 | 0.0051 | 0.0050 | 0.0001 | 0.7068 |
| Immune_system | 0.0034 | 0.0034 | 0.0035 | 0.0034 | 0.0001 | 0.3084 |
| Aging | 0.0034^a^ | 0.0033^b^ | 0.0033^b^ | 0.0033^ab^ | 0.0001 | 0.0482 |
| Endocrine_and_metabolic_diseases | 0.0029 | 0.0029 | 0.0029 | 0.0028 | 0.0001 | 0.3815 |
| Nervous_system | 0.0030 | 0.0029 | 0.0024 | 0.0029 | 0.0001 | 0.4354 |
| Environmental_adaptation | 0.0016 | 0.0017 | 0.0018 | 0.0017 | 0.0003 | 0.3525 |
| Signaling_molecules_and_interaction | 0.0023 | 0.0022 | 0.0020 | 0.0023 | 0.0001 | 0.3646 |
| Cardiovascular_diseases | 0.0014 | 0.0014 | 0.0014 | 0.0014 | 0.0001 | 0.9798 |
| Neurodegenerative_diseases | 0.0007 | 0.0007 | 0.0007 | 0.0007 | 0.0001 | 0.0801 |
| Digestive_system | 0.0012 | 0.0012 | 0.0011 | 0.0013 | 0.0001 | 0.3736 |
| Viral_protein_family | 0.0007 | 0.0007 | 0.0007 | 0.0007 | 0.0001 | 0.2454 |
| Immune_diseases | 0.0003 | 0.0003 | 0.0003 | 0.0003 | 0.0001 | 0.6703 |
| Excretory_system | 0.0002 | 0.0001 | 0.0001 | 1.0000 | 0.0001 | 0.2496 |
| Substance_dependence | 0.0001 | 0.0001 | 0.0001 | 0.0001 | 0.0001 | 0.4470 |
| Circulatory_system | 0.0000 | 0.0000 | 0.0000 | 0.0000 | 0.0001 | 0.3181 |


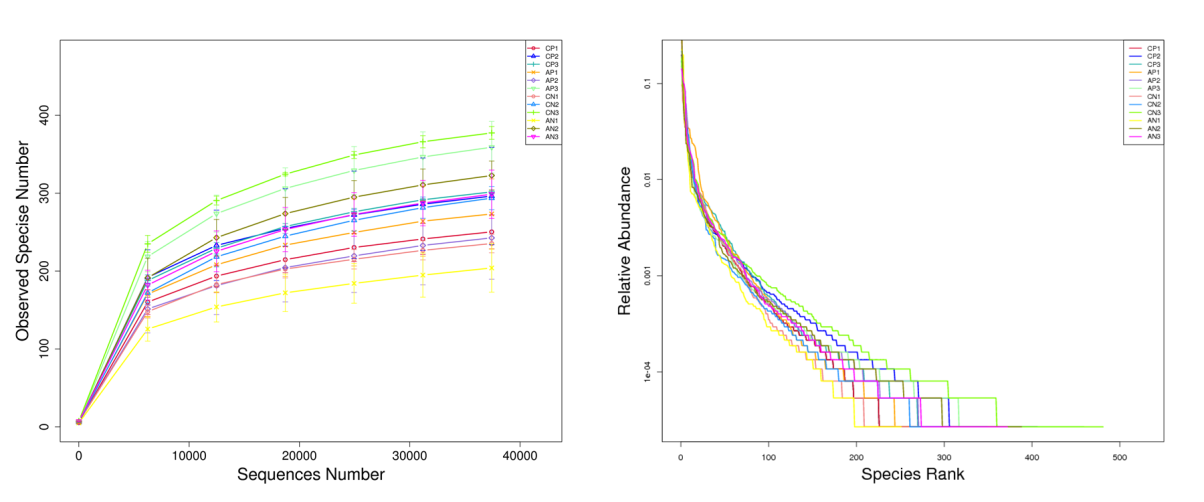


**Figure S2** The Alpha diversity curves of Rarefaction and Rank abundance. Abbreviation: CN, CON group without *S. ser. Enteritidis*-infection; AN, APS group (500 mg/kg diet) without *S. ser. Enteritidis*-infection; CP, CON group with the *S. ser. Enteritidis*-infection. AP, APS supplementation and *S. ser. Enteritidis*-infection.


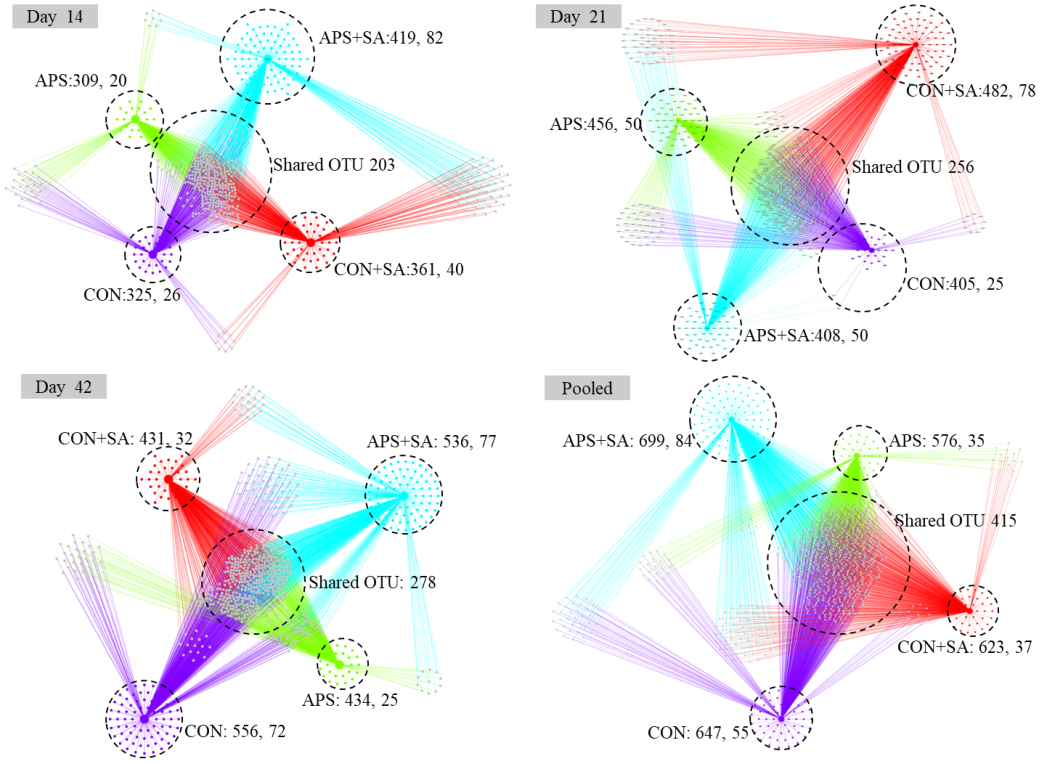


**Figure S3** The OTU numbers of the treatments in different growing ages of broilers were made using Cytoscape (3.8.2), <https://cytoscape.org/>. The numbers for each group addressed the total OTU amounts and special OTU numbers in each group. The shared OTU represented the OTU numbers across the four groups. Abbreviations: CON; control group in the pair-fed groups, APS; APS supplemented group in the pair-fed groups, CON+SA; control group in the *S. ser. Enteritidis*-challenged groups, APS+SA; APS supplemented group in the *S. ser. Enteritidis*-challenged groups.


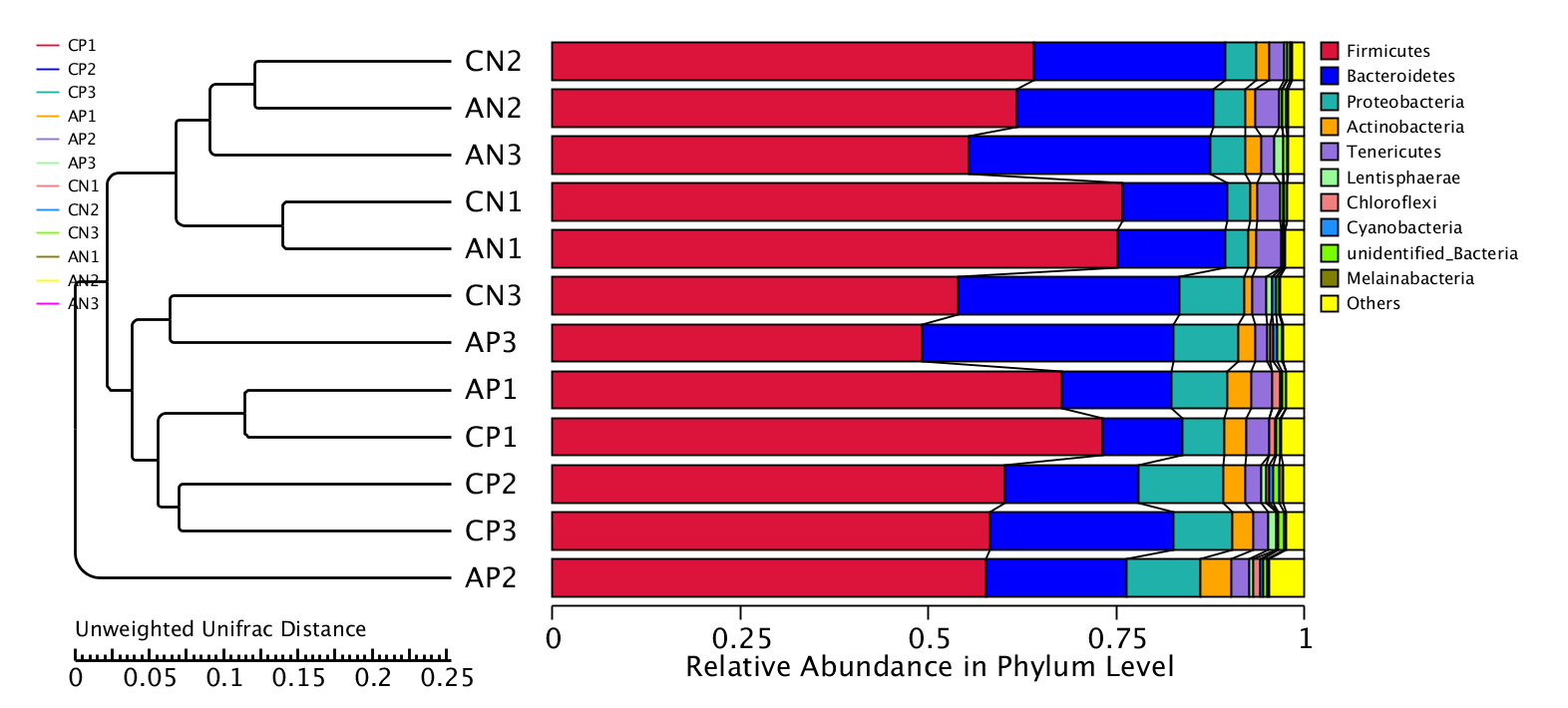


**Figure S4** The Unweighted Unifrac distances analysis and comparison among different groups. The microbial community structures in different ages (day 14, 21, and 42) of the CON, APS, CON+SA, and APS+SA groups were almost separated in the hierarchical clustering tree. Abbreviation: CN, CON group without *S. ser. Enteritidis*-infection; AN, APS group without *S. ser. Enteritidis*-infection; CP, CON group with the *S. ser. Enteritidis*-infection. AP, APS supplementation and *S. ser. Enteritidis*-infection.


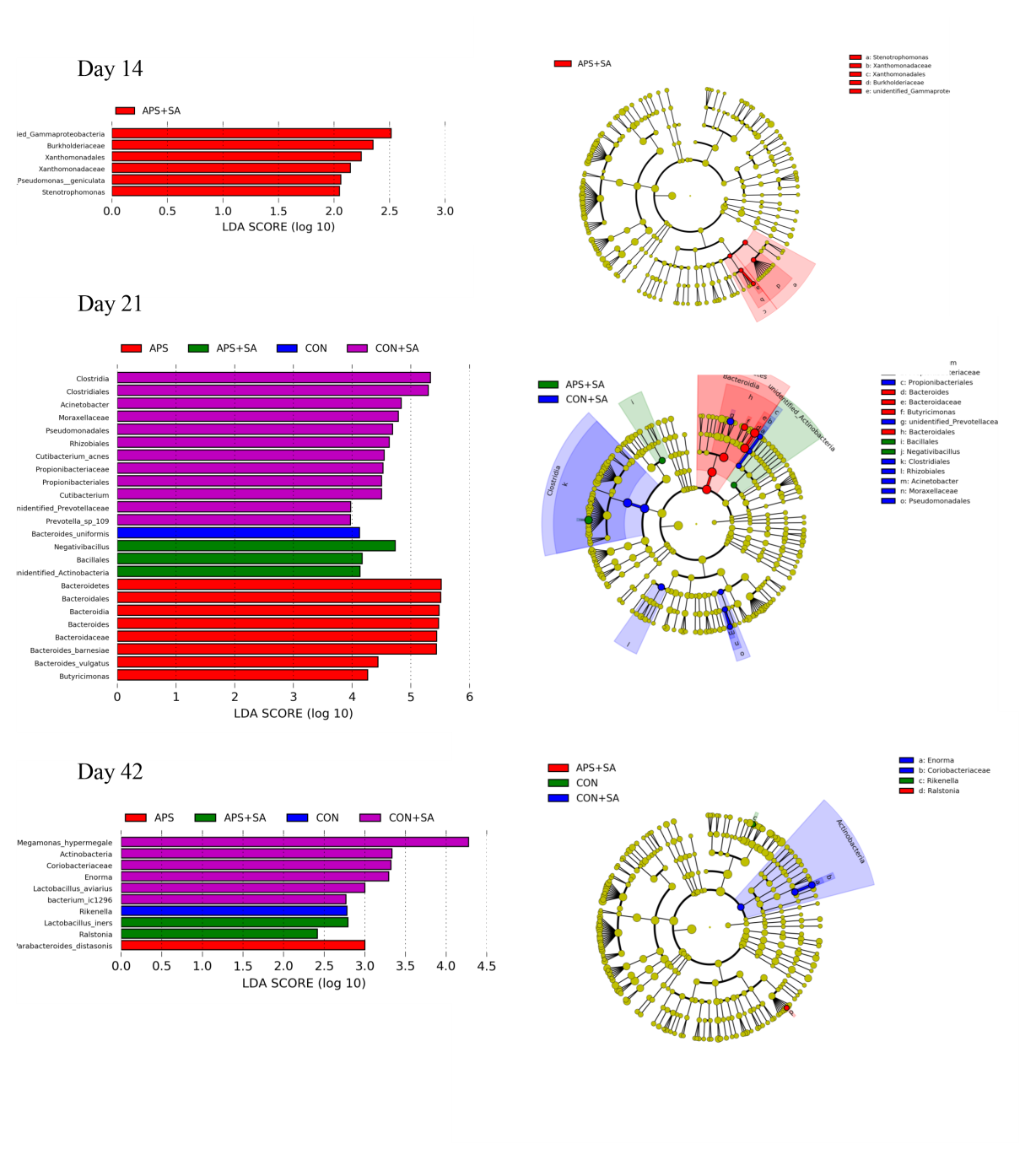


**Figure S5** LefSe analysis of cecal microbial community in broilers from CON, APS, CON+SA and APS+SA groups on day 14, 21, and 42. The histogram displayed those species with significant difference that have an LDA score greater than the estimated value with the default score 2. The length of the bar represents the LDA score indicating the difference of species in the four groups. The cladogram showed those microbial species with significant differences in CON, APS, CON+SA and APS+SA groups. Red, green, and blue indicate different groups, with the species classification at the level of phylum, class, order, family, and genus shown from the inside to outside. The red, green, and blue nodes in the phylogenetic tree represent microbial species that play important roles in the APS+SA, CON, and CON+SA groups, respectively. Yellow nodes represent species with no significant difference. Abbreviations: CON, control pair-fed group; APS, APS-supplemented pair-fed group; CON+SA, control *S. ser. Enteritidis*-challenged group; APS+SA, APS-supplemented *S. ser. Enteritidis*-challenged group; *SA+*, *S. ser. Enteritidis*-challenged groups; *SA-*, pair-fed groups.
